# Hypoxia promotes glioma-associated macrophage infiltration via periostin and subsequent M2 polarization by upregulating TGF-beta and M-CSFR

**DOI:** 10.18632/oncotarget.11825

**Published:** 2016-09-02

**Authors:** Xiaofan Guo, Hao Xue, Qianqian Shao, Jian Wang, Xing Guo, Xi Chen, Jinsen Zhang, Shugang Xu, Tong Li, Ping Zhang, Xiao Gao, Wei Qiu, Qinglin Liu, Gang Li

**Affiliations:** ^1^ Department of Neurosurgery, Qilu Hospital of Shandong University, Jinan, Shandong Province, P.R. China; ^2^ Brain Science Research Institute, Shandong University, Jinan, Shandong Province, P.R. China; ^3^ Institute of Basic Medical Sciences and Key Laboratory of Cardiovascular Proteomics of Shandong Province, P.R. China; ^4^ Department of Biomedicine, University of Bergen, Bergen, Norway; ^5^ Department of Neurosurgery, Dezhou People's Hospital, Dezhou, Shandong Province, P.R. China

**Keywords:** glioma, hypoxia, tumor-associated macrophage, M2 macrophage, acriflavine

## Abstract

Tumor-associated macrophages (TAMs) are enriched in gliomas and help create a tumor-immunosuppressive microenvironment. A distinct M2-skewed type of macrophages makes up the majority of glioma TAMs, and these cells exhibit pro-tumor functions. Gliomas contain large hypoxic areas, and the presence of a correlation between the density of M2-polarized TAMs and hypoxic areas suggests that hypoxia plays a supportive role during TAM recruitment and induction. Here, we investigated the effects of hypoxia on human macrophage recruitment and M2 polarization. We also investigated the influence of the HIF inhibitor acriflavine (ACF) on M2 TAM infiltration and tumor progression *in vivo*. We found that hypoxia increased periostin (POSTN) expression in glioma cells and promoted the recruitment of macrophages. Hypoxia-inducible POSTN expression was increased by TGF-α via the RTK/PI3K pathway, and this effect was blocked by treating hypoxic cells with ACF. We also demonstrated that both a hypoxic environment and hypoxia-treated glioma cell supernatants were capable of polarizing macrophages toward a M2 phenotype. ACF partially reversed the M2 polarization of macrophages by inhibiting the upregulation of M-CSFR in macrophages and TGF-β in glioma cells under hypoxic conditions. Administering ACF also ablated tumor progression *in vivo*. Our findings reveal a mechanism that underlies hypoxia-induced TAM enrichment and M2 polarization and suggest that pharmacologically inhibiting HIFs may reduce M2-polarized TAM infiltration and glioma progression.

## INTRODUCTION

Gliomas are the most common brain-derived neoplasms [1]. Glioblastomas (GBM, WHO grade IV glioma) carry the worst prognosis, and GBM patients have a median survival time of less than 16 months even when treated [2]. The recalcitrance of malignant gliomas to standard therapies is believed to result from their heterogeneity and the unique tumor microenvironment which contains multiple types of cells, including tumor cells, fibroblasts, and various types of immune cells [3].

Two polarized macrophage phenotypes have been identified, including classically activated macrophages (M1 type) and alternatively activated macrophages (M2 type) [4]. M1 and M2 macrophages are believed to limit and promote tumor progression, respectively [5, 6]. TAMs are a group of immune cells that reside in and around the tumor microenvironment and are more likely to be M2 macrophages [7]. Circulating monocytes or resident microglia (in the CNS) are drawn to enter to tumor region [8] by chemotactic factors and are subsequently induced to differentiate into TAMs in the tumor microenvironment. CD11b is commonly used as a general cell surface marker for all TAMs [9]. Several surface markers and cytokines, such as CD163, CD206, IL-10, IL-1ra, and CCL-22, are used to identify M2 type TAMs, whereas factors including IL-6, TNF-alpha, IL-12, IL-23, and IL-1b have been suggested as markers for M1 type TAMs [10–12]. TAMs are thought to be the most abundantly infiltrating immune cells in gliomas, especially GBM [3, 13], and they promote glioma growth and invasion. Novel therapies that target the immune environment have already shown some promise as treatments for glioma [14–17].

Rapidly growing solid tumors, such as gliomas, routinely outstrip the oxygen supply provided by surrounding capillary vessels, and this results in hypoxia. Given that macrophages are often retained in tumors as an immature cell type [18, 19] and the fact that macrophages preferentially accumulate in hypoxic areas, where they polarize into a specific cell type [20–22], hypoxia may help to promote the recruitment and re-specification of TAMs. However, the mechanisms underlying these processes have not yet been fully defined in glioma.

POSTN is a cell-secreted adhesion protein that was originally isolated as an osteoblast and mesenchyme-specific factor [23]. A number of recent studies have shown that POSTN is capable of promoting tumor progression in many cancers [24–26]. POSTN has been shown to perform a novel function by acting as a potent attractant of TAMs in GBM [27]. Interestingly, POSTN was reported to be increased under hypoxic conditions in non-small cell lung cancer, an effect that promoted tumor cell survival [28]. The question of whether hypoxia increased POSTN to promote the aggregation of M2 type TAMs in gliomas attracted our interest.

In a hypoxic state, cellular responses to O_2_ deprivation are largely mediated by hypoxia-inducible factors (HIFs) [29]. When stabilized by hypoxia, HIF subunits translocate into the nucleus and bind to hypoxia-response elements (HREs) to promote the expression of specific genes [30, 31]. HIFs accumulate in both neoplastic and inflammatory cells within the tumor microenvironment, and they have been shown to promote progression in a variety of cancers [32, 33]. ACF, a viable source for future anti-cancer drugs, is a mixture of proflavine and trypaflavin that inhibits dimerization between HIF-α and ARNT and thereby blocks the effects of HIF [34, 35]. Treatment with ACF impairs tumor progression, and, interestingly, decreases TAM infiltration in patients with breast or colorectal cancer [35, 36]. However, it is not yet known whether ACF is capable of reducing the hypoxia-inducible infiltration of M2 TAMs in gliomas.

In this study, we investigated the effect of hypoxia on TAM infiltration and the mechanism underlying this relationship. Furthermore, we explored the effect of the hypoxic tumor microenvironment on the polarization of TAMs and examined whether ACF interferes with the hypoxia-driven recruitment and polarization of TAMs within gliomas. We demonstrate that hypoxia increased the expression of POSTN via the TGF-α/RTK/PI3K pathway, which resulted in the recruitment of more TAMs to HIF-1α-positive regions. TAMs are located close to perivascular niches in gliomas with low HIF-1α expression, and their distribution became more dispersed in gliomas with high HIF-1α expression. In addition, we found that in gliomas, hypoxia promoted M2 polarization in TAMs and that ACF was capable of partially reversing the effects of hypoxia on the recruitment and M2 polarization of TAMs. Administering ACF also ablated tumor progression *in vivo*.

## RESULTS

### Hypoxia, POSTN expression and TAM infiltration are associated with glioma grade and prognosis

**Figure 1:**
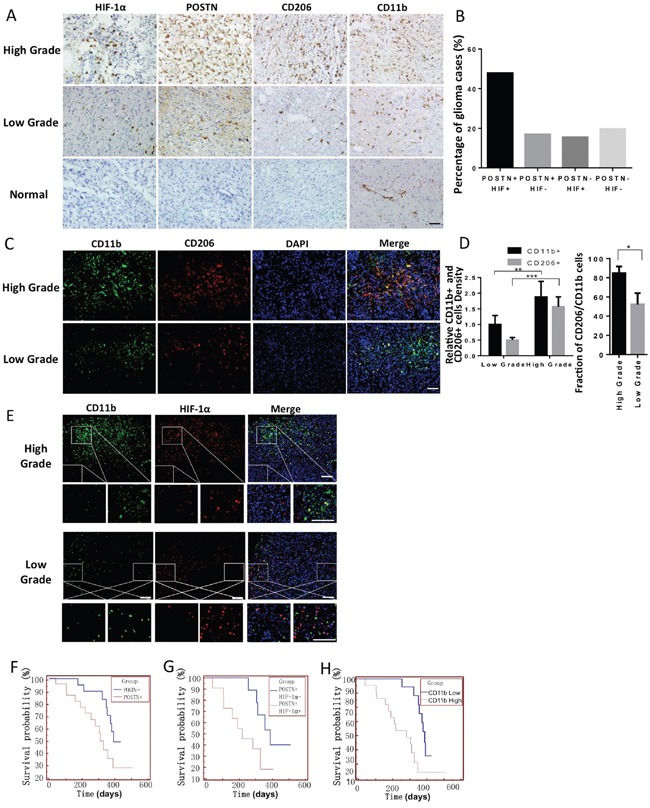
Hypoxia, POSTN expression, TAM infiltration and the proportion of M2 subtype TAMs increased as the grade of glioma increased, and all were correlated with a worse prognosis **A.** IHC staining for POSTN, HIF-1α, CD11b and CD206 in four consecutive tissue slides obtained from gliomas with different grades and normal brain tissue specimens. Scale bars, 50 μm. **B.** A graphical analysis of (A). In all, 47.8% of the glioma cases showed POSTN+ and HIF-1α+ staining, and 16.9% and 15.5% of the GBM cases showed single-positive cells that were POSTN+/HIF-1α- or POSTN-/HIF-1α+, respectively. Only 19.8% of the cases showed both types of negative staining. **C.** Immunofluorescence staining for the TAM marker CD11b (green) and the M2 type TAM marker CD206 (red). **D.** A graphical analysis of figure (C) showing that TAM infiltration and the proportion of M2 type cells increased as the grade of the glioma increased. TAM density was analyzed using ImageJ. *, P <0.05; **, P <0.01; ***, P <0.001 (n =5 tumors; mean ± s.e.m.; two-tailed unpaired t-test). **E.** Immunofluorescence analysis of CD11b (green) and HIF-1α (red) expression in glioma tissue slides showing that TAMs are enriched in HIF-1α-abundant regions. The areas indicated in the squares are enlarged and shown under each picture. Scale bars, 200 μm. **F** and **G.** Kaplan-Meier curves analysis of 22 glioma patients with positive POSTN staining and 20 patients with negative POSTN staining showing that the POSTN+ group had a worse prognosis (median survival: 398 days and 311 days, respectively, Log Rank test, p<0.05). In addition, among the 22 POSTN-positive patients, the HIF-1α-positive patients had even worse prognoses (median survival: 378 days vs 218 days, Log Rank test, p<0.05). **H.** Glioma patients with high CD11b+ TAM infiltration in their tumors had shorter postsurgical survival times than those with low CD11b expression (median survival: 391 days vs 311 days, Log Rank test, n=42, p<0.05, the patients' information was shown in Supplementary Table S1).

To explore the relationships between hypoxia, POSTN expression and the infiltration of TAMs in gliomas, we performed immunohistochemical (IHC) staining on sections obtained from different grade gliomas. We found that HIF-1α expression, POSTN expression, and the infiltration of TAMs (CD11b+) and M2 type TAMs (CD206+) increased as the grade of the glioma increased (Figure 1A). In addition, HIF-1α+/POSTN+ patients accounted for 47.8% of the 42 patients, suggesting a potential association between POSTN and hypoxia (Figure 1B). Immunofluorescence staining for CD11b and CD206 in different grade glioma sections also indicated that TAM infiltration increased and that there was a higher proportion of the M2 subtype TAMs in higher grade gliomas (Figure 1C, 1D). Immunofluorescence staining for HIF-1α and CD11b indicated that the density of TAMs was increased in HIF-1α-positive regions (Figure 1E). A correlation between the distribution of POSTN and CD206+ TAMs was also found in gliomas with different grades (Supplementary Figure S1). These findings support the notion that there is a positive correlation between hypoxia, POSTN expression and TAM infiltration in gliomas.

To further examine the impact of hypoxia, POSTN expression and TAM infiltration on cancer development, we used Kaplan-Meier estimates to compare survival rates between patients according to their IHC results. The pathological and general characteristics of the patients who participated in our analysis are listed in Table S1. This analysis demonstrated that POSTN-positive patients had shorter postoperative survival times than POSTN-negative patients (Figure 1F) and that double-positive patients (POSTN+/HIF-1α+) had even shorter survival times (Figure 1G). These findings indicated that both hypoxia and POSTN expression represent potential prognostic biomarkers in gliomas. Moreover, TAMs also play critical roles in glioma progression. The median postsurgical survival time in patients with high CD11b+ TAM infiltration was significantly lower than in patients with low CD11b+ expression (Figure 1H).

It is critical to identify the main source of TAMs in gliomas. It has been demonstrated that microglia in the CNS exhibit a CX3CR1+/CCR2− phenotype, whereas monocyte-derived macrophages are CX3CR1-/CCR2+ [37, 38]. We therefore used these markers to determine the origin of the TAMs in human gliomas. We analyzed 17 glioma (9 GBM, 4 astrocytoma, and 4 anaplastic astrocytoma) and 4 normal brain tissue samples. The results demonstrated that in normal brains, most macrophages were CX3CR1+ (a microglial marker), implying that they were resident microglia. Only 1 of the 4 normal brain tissues stained weakly positive for CCR2+ (monocyte-derived macrophages). Next, we found that TAMs in human gliomas were mainly CCR2+/CX3CR1−, indicating that they were monocyte-derived macrophages (Supplementary Figure S2A, S2B). CCR2+ cells were detected in all 17 glioma tissue samples, and CX3CR1+ cells were found in 12 of the 17 glioma tissue samples. These results are in accordance with the results of a previous report by Bao et al [27].

Because hypoxia and POSTN are associated with the enrichment of TAMs in gliomas, we next sought to determine whether the hypoxia-induced upregulation of POSTN expression attracted more TAMs and subsequently promoted glioma progression.

### POSTN expression was increased by hypoxia and was potentially capable of recruiting macrophages

**Figure 2:**
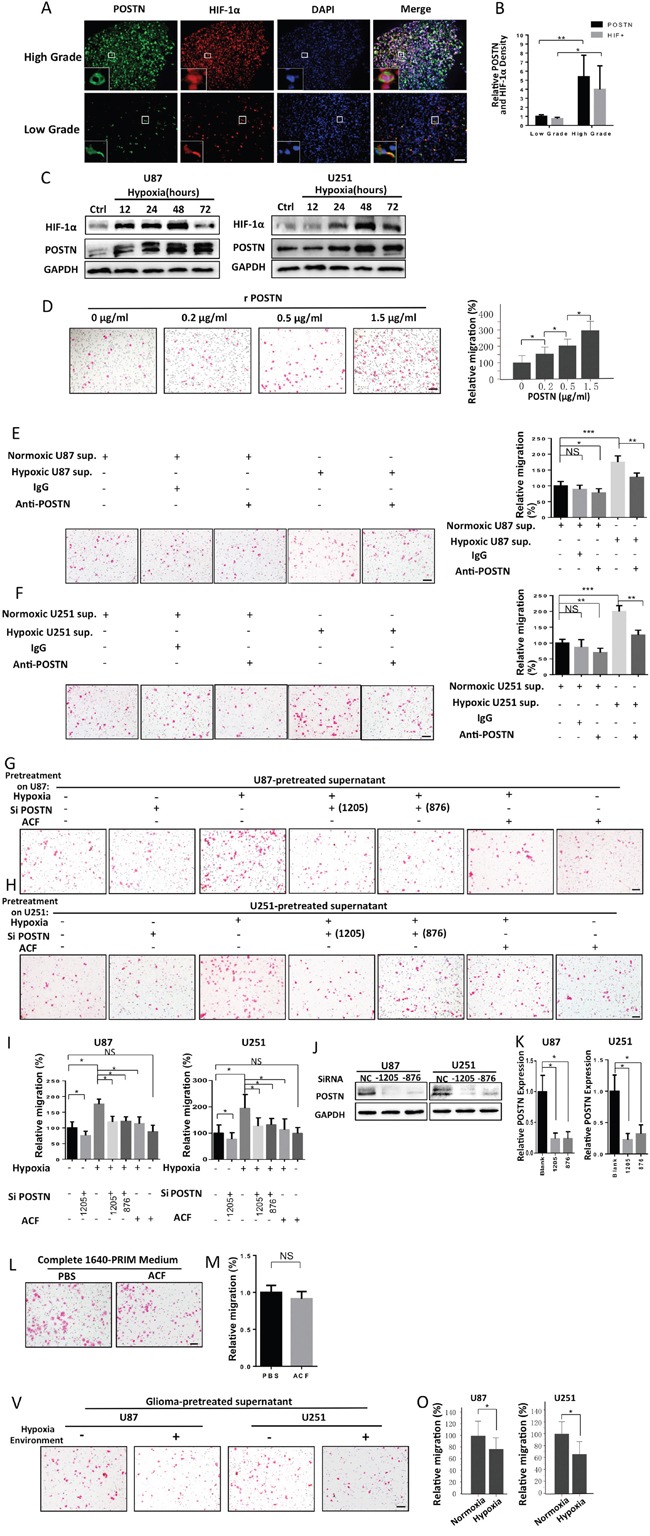
Hypoxia-inducible POSTN secretion by glioma cells promotes the recruitment of macrophages **A.** Immunofluorescence staining for POSTN (green) and HIF-1α (red). Scale bars, 100 μm. **B.** A graphical analysis of figure (A) showing that both HIF-1α and POSTN expression increased as the grade of the glioma increased. HIF-1α and POSTN expression was analyzed with ImageJ. *, P <0.05; **, P <0.01 (n =5 tumors; mean ± s.e.m.; two-tailed unpaired t-test). **C.** Western blot analysis of POSTN and HIF-1α levels in U87 and U251 cells that were exposed to hypoxia showing that they increased over the time-course. **D.** Representative images of THP-1 macrophage-like cells migrating towards different concentrations of rPOSTN in transwell assays. Scale bar, 100 μm. Graphical analysis of (D)showing that THP-1 induced macrophages to migrate towards rPOSTN in a dose-dependent manner. **E** and **F.** Representative images of THP-1 macrophage-like cells migrating towards normoxia- or hypoxia- treated U87 or U251 cell culture supernatants pre-incubated with or without anti-POSTN antibodies. A graphical analysis of (E) and (F) shows that the increased macrophages migration toward hypoxia-treated glioma cell supernatants was attenuated by the application of anti-POSTN antibodies. Scale bar, 100 μm. **G** and **H.** Representative images of THP-1 macrophage-like cells migrating towards different conditioned culture supernatants that were obtained from U87 and U251 cells. **I.** A graphical analysis of (E) and (F) showing that the increased migration observed in THP-1 induced macrophages toward hypoxia-treated U87 and U251 cell supernatants was reversed by transfecting the cells with siPOSTN before the induction of hypoxia. Moreover, pretreatment of U87 or U251 cells with ACF during exposure to hypoxia also eliminated this trend. **J.** Western blot analysis of POSTN levels in U87 or U251 cells that were transfected with siPOSTN (1205 or 876) or vector. **K.** A graphical analysis of (J) showing that there was a significant decrease in POSTN expression in the siPOSTN groups in both U87 and U251 cells. The relative POSTN expression level was determined using ImageJ software. The results are shown as the means ± s.e.m. (n = 3; *, P <0.05; two-tailed unpaired t-test). **L.** Representative images of THP-1 macrophages migrating toward complete PRIM-1640 medium in the presence of PBS or ACF (3 μM). **M.** A graphical analysis of (L) showing that ACF had no direct effect on macrophage migration. **V.** Representative images of THP-1 macrophages migrating towards the conditioned medium of U87/U251 cells when exposed to a normoxic or hypoxic environment. **O.** A graphical analysis of (V) showing that the migratory activity of macrophages was impaired by hypoxia. All transwell assays were repeated three times. *, P <0.05; **, P <0.01; NS, P >0.05 (n = 5 fields, mean ± s.e.m., two-tailed unpaired t-test).

To investigate the connection between hypoxia and POSTN production, we performed immunofluorescence staining for POSTN and HIF-1α in sections from different grade gliomas. Our results confirm that both HIF-1α and POSTN expression increased as the grade of the glioma increased (Figure 2A, 2B). Moreover, HIF-1α and POSTN were observed to coexpress within the same cell at higher magnification. To determine whether hypoxia induces an increase in the expression of POSTN at the cellular level, we next exposed U87 and U251 cells to a hypoxic environment and then examined their POSTN expression level. The results showed that hypoxia caused a significant increase in POSTN expression and that the expression of POSTN was maintained at high levels even at 3 days after hypoxia stimulation (Figure 2C). The level of HIF-1α did not increase after day 2, indicating that 2 days of hypoxia stimulation was capable of inducing the maximum level of HIF-1α expression.

To determine whether the hypoxia-inducible expression of POSTN in glioma cells is capable of attracting macrophages, we performed a series of migration and invasion assays. The macrophage-like cells used in these migration experiments were induced from THP-1 cells by stimulating THP-1 cells with PMA for 48 h. We first tested the impact of different concentrations of recombinant POSTN (rPOSTN) on macrophage migration. The THP-1 macrophages exhibited more migration toward rPOSTN as the rPOSTN level incrementally increased (Figure 2D). We then compared the chemotactic properties displayed by normoxia- and hypoxia-treated glioma cell supernatants. We found that hypoxia-treated glioma cell supernatants elicited a stronger chemotactic effect. However, pre-incubating hypoxia-stimulated U87 or U251 glioma cells in culture media containing an anti-POSTN antibody attenuated this macrophage migration-promoting effect (Figure 2E, 2F). Moreover, this hypoxia-inducible effect was impaired when POSTN expression was silenced in U87/U251 cells or when a HIF inhibitor (ACF) was added to the culture medium of the glioma cells during hypoxia stimulation (Figure 2G-2I). To eliminate the impact of ACF itself on the migration of THP-1 macrophages, we tested macrophage migration after we added either ACF (3μM) or PBS to the medium, and we found that there was no significant difference in the rate of migration between the two groups (Figure 2L, 2M). Finally, we explored the impact of a hypoxic environment on macrophage migration. Interestingly, we found that the macrophage motility was attenuated by hypoxia (Figure 2V, 2O). This phenomenon partially explains the mechanism by which TAMs become trapped in hypoxic areas after they are initially attracted to them. Collectively, these data demonstrate that hypoxia-inducible POSTN expression enhanced the chemotactic effect of glioma cells on macrophages.

### TAM infiltration is associated with perivascular niches

Glioma stem-like cells (GSLCs) are a group glioma cells that are able to self-renew, differentiate and repopulate a tumor [39, 40]. GSLCs have been reported to reside in the perivascular niches of gliomas [39, 41]. A recent study showed that in gliomas, POSTN is mainly secreted by GSLCs [27]. In gliomas, an additional two important TAM chemotactic factors, SDF-1α [42] and osteopontin (OPN) [43], have also been reported to be expressed in these niches [44]. Interestingly, several recent publications have demonstrated that perivascular niches are located in hypoxic areas [44–46]. Whether hypoxia is capable of affecting the expression of chemotactic factors in perivascular niches to subsequently influence the recruitment of TAMs intrigued us.

To explore the connection between POSTN expression, GSLCs and hypoxia in gliomas, we examined the POSTN expression pattern in human GBM surgical specimens that exhibited different levels of HIF-1α. We found that in gliomas that expressed low levels of HIF-1α, 58.8% of the POSTN+ cells were CD133+ GSLCs. However, in the glioma specimens, as the level of HIF-1α expression increased, we observed a corresponding increase in the number of POSTN+ cells in non-stem glioma cells (NSGCs) (CD133-) (Supplementary Figure S3A, S3B). These data imply that POSTN is expressed mainly in GSLCs in gliomas with low levels of hypoxia. However, as the level of HIF-1α increased, more NSGCs begin to secrete POSTN, and this attracted more TAMs to the hypoxic area.

We next performed several immunofluorescence staining experiments in both low-and high-hypoxic glioma specimens to evaluate the relationship between TAM recruitment and perivascular niches. Because the endothelium is also an important part of the GSLC niche [39], we used CD31 (an endothelial cells marker) to help us to localize these niches. We found that in low HIF-1α expressing glioma specimens, some HIF-1α expression was localized in and around the blood vessel walls in perivascular niches (Supplementary Figure S3D). The HIF-1α expression domain became more disperse in glioma specimens with high levels of hypoxia (Supplementary Figure S3D, S3E). Immunofluorescence staining showed that GSLCs (CD133+) were located significantly closer to tumor perivascular areas in glioma tissues. No significant difference was found in the distribution or proportion of GSLCs between low and high HIF-1α-expressing glioma sections (Supplementary Figure S3F). We observed that POSTN+ cells were located closer to perivascular niches in low HIF-1α glioma specimens. The high rate of POSTN expression in GSLCs in low HIF-1α glioma specimens may explain this POSTN distribution pattern. However, an increase in the production of POSTN and a more diffuse distribution of POSTN were observed in both perivascular and non-vascular areas in the high HIF-1α expressing glioma specimens (Supplementary Figure S3G). Next, we analyzed the localization of the chemokine SDF-1α and OPN in glioma sections. Both SDF-1α and OPN were found to be localized in perivascular areas, and portions of their staining patterns overlapped with perivascular niches in both low and high HIF-1α GBM sections (Supplementary Figure S3H and S3I). The relative densities of SDF-1α and OPN labeling were 1.3- and 2.1-fold higher, respectively, in high HIF-1α gliomas than in low HIF-1α glioma sections. However, the distribution of these two molecules was not significantly altered in low and high HIF-1α expressing gliomas. The hypoxia-inducible increase in their expression was much smaller than that of POSTN, which was 5.2–fold higher in high-HIF-1α GBM sections compared with low HIF-1α GBM specimens (Supplementary Figure S3K-S3M). We next examined the localization of TAMs using CD11b and CD31. TAMs were enriched in perivascular areas in low HIF-1α glioma sections, but their distribution became more diffuse when the HIF-1α positive area grew in size (Supplementary Figure S3J). These data demonstrate that in gliomas, GSLCs, SDF-1α and OPN expression aggregate in perivascular niches. Both POSTN expression and TAM infiltration were concentrated in perivascular areas in low HIF-1α glioma tissues, but their distribution became more disseminated as the expression of HIF-1α increased.

### Hypoxia-inducible TGF-α enhances the expression of POSTN via the RTK/PI3K pathway in U87 and U251 cells grown under hypoxic conditions

**Figure 3:**
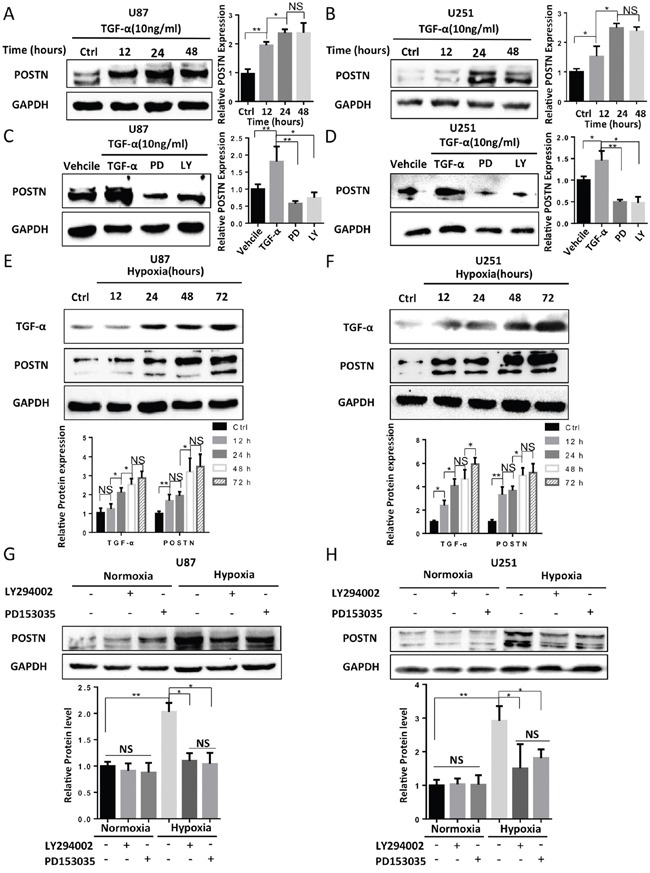
Hypoxia increased the secretion of TGF-α, which subsequently enhanced the expression of POSTN via the RTK/PI3K pathway in U87 and U251 cells **A** and **B.** U87 and U251 cells were treated with 10 ng/ml TGF-α for 12, 24 and 48 h. The protein expression level of POSTN was then analyzed (one way Anova test). **C** and **D.** U87 and U251 cells were pretreated with vehicle (DMSO), PD153035 (10 μM) or LY294002 (10 μM) for 1 h before TGF-α was added for 48 h. POSTN expression was then analyzed (two-tailed unpaired t-test). **E** and **F.** TGF-α and POSTN expression were increased by hypoxia stimulation in a time-dependent manner. The expression of TGF-α and POSTN were analyzed (one way Anova test). **G** and **H.** The hypoxia-induced upregulation of POSTN was attenuated by pretreating the cells with the signaling pathway inhibitors LY294002 (10 μM) or PD153035 (10 μM) for 1 h before the cells were exposed to normoxic or hypoxic conditions (two-tailed unpaired t-test). The results are shown as the means ± s.e.m, n = 3. The protein expression levels were analyzed with ImageJ. *, P <0.05; **, P <0.01 ***, P <0.001; NS, P>0.05.

Many pathways and molecules have been reported to increase POSTN expression under hypoxic conditions in different types of cells [28, 47]. We treated U87 and U251 cells with TGF-α, which is a hypoxia-inducible growth factor known to lie downstream of HIF-1α, and we found that POSTN expression was elevated in a time-dependent manner (Figure 3A, 3B). Next, we pretreated U87 and U251 cells with PD153035, a selective inhibitor of the epidermal growth factor receptor (EGFR) tyrosine kinase, or LY294002, a PI3-K inhibitor, prior to stimulation with TGF-α. The results showed that both PD153035 and LY294002 significantly blocked the increase in POSTN expression that was normally induced by TGF-α (Figure 3C, 3D). These results suggest that TGF-α enhanced the expression of POSTN via the RTK/PI3K pathway. To determine whether the RTK/PI3K pathway is involved in hypoxia-inducible POSTN expression, we first examined the time courses of TGF-α and POSTN expression in cells stimulated with hypoxia. As shown in Figure 3E and 3F, the expression of both TGF-α and POSTN were enhanced by hypoxia. Next, signaling inhibitors were applied to analyze their effect on hypoxia-inducible POSTN expression. The results revealed that inhibiting either RTK or PI3K decreased POSTN expression in U87 and U251 cells exposed to hypoxia (Figure 3G, 3H). Collectively, our findings demonstrate that in glioma cells, hypoxia-induced increases in POSTN expression are mediated via the TGF-α/RTK/PI3-K pathway.

### Human monocyte-derived macrophage (HMDM) morphologies and phenotypes vary when cells are exposed to different hypoxic conditions

**Figure 4:**
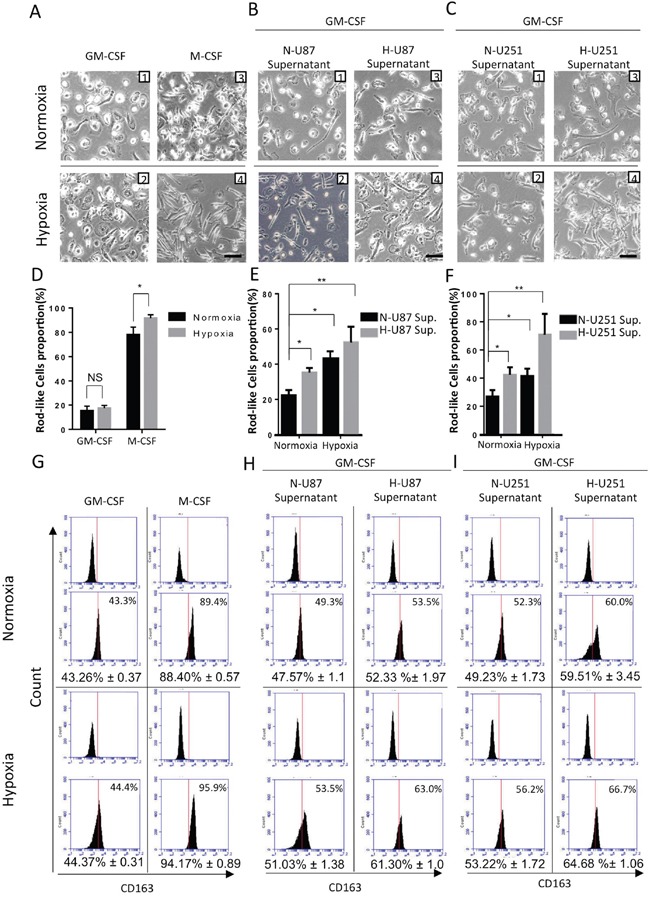
Changes in cell morphology and surface markers in HMDMs exposed to different hypoxic conditions We refer to the two hypoxic conditions using the following abbreviations: N+N-U87 sup., macrophages were exposed to a normoxic environment and normoxia-treated U87 cell culture supernatants; and H+H-U87 sup., macrophages were exposed to a hypoxic environment and hypoxia-treated U87 cell culture supernatants. **A.** Representative images of human monocytes that were cultured for 7 days with M-CSF or GM-CSF under normoxic or hypoxic conditions. Scale bars, 50 μm. **B.** Representative images of human monocytes that were cultured for 7 days with GM-CSF in the presence of N+N-U87 sup., N+H-U87 sup., H+N-U87 sup. or H+H-U87 sup. Scale bars, 50 μm. **C.** The same protocol was followed to culture human monocytes with U251 cell supernatants. Scale bars, 50 μm. **D.** A graphical analysis of (A) showing that hypoxia increased the proportion of rod-like macrophages in cell cultures that were stimulated using M-CSF. However, the percentage of rod-like cells in the macrophages that were induced using GM-CSF was not significantly changed whether or not the cells was stimulated with hypoxia. **E** and **F.** A graphical analysis of (B and C) showing that both a hypoxic environment and hypoxia-conditioned U87/U251 cell supernatants were able to increase the percentage of rod-like HMDMs. *, P <0.05. NS, P >0.05 (n =3 healthy donor MDMs, 5 fields were used to calculate each group, and the results are shown as the mean ± s.e.m., two-tailed paired t-test). **G-I.** Phenotype switching in HMDMs exposed to the different hypoxic stimulations mentioned in (A-C) was evaluated using flow cytometry. The surface marker CD163 was selected as the M2 polarized macrophage marker. Results are shown from a representative experiment. The numbers in parenthesis represent the percentage of M2 macrophages that was obtained during flow cytometric analysis of macrophages obtained from 3 patients. The data are presented as the mean ± s.e.m.; n=3.

We next sought to investigate the impact of hypoxic conditions in gliomas on the polarization of TAMs using a HMDM. Because hypoxia and tumor supernatants are not enough to induce monocytes to differentiate into macrophages, the M1/M2 HMDM model was used in our experiments, and GM-CSF and M-CSF were used to induce macrophages. ELISA and RT-qPCR analyses of macrophage markers showed that HMDMs induced by M-CSF expressed much higher levels of M2 macrophage markers (e.g., TGF-β, IL-1ra, CD163, IL-10 and CCL-22) and lower levels of M1 markers (e.g., IL-12, IL-23, IL-1b, IL-6 and TNF-α) than GM-CSF stimulated HMDMs (Supplementary Figure S4A-S4J). Flow cytometry analysis similarly showed that 89.4% of the HMDMs were M2 polarized (CD163+) after the cells were stimulated with M-CSF (Figure 4G). The GM-CSF-induced M1 subtype macrophages showed a primarily round morphology, whereas the M-CSF-induced M2 subtype macrophages were predominantly spindle-like (Figure 4A1, 4A3). However, not all of the monocytes transformed in the same direction. We observed that approximately 84.6% of the round cells and 78.0% of the spindle-like cells were transformed in the presence of GM-CSF and M-CSF, respectively (Figure 4A, 4D). The proportion of spindle-like cells was used as an indicator of M2 polarization in our experiment.

Next, we sought to determine whether hypoxia, in the absence of tumor supernatants, is capable of polarizing HMDMs. We cultured human monocytes for 7 days under normoxic or hypoxic conditions in the presence of GM-CSF or M-CSF. We found that hypoxia did not alter the rate of spindle-like cell transformation or the proportion of CD163+ cells in the GM-CSF group, but it did increase the proportion of spindle-like cells in the M-CSF group from 78.0% to 91.6% (Figure 4A, 4D). The proportion of CD163+ cells increased from 88.4% to 94.93% (Figure 4G).

In the following experiments, we applied different glioma cell culture supernatants into the macrophages polarization experiment. We sought to determine whether a hypoxic environment (in the presence of glioma culture supernatant) or hypoxia-treated glioma cell supernatants or both would alter polarization in TAMs. We added either normoxia- or hypoxia-treated U87/U251 cell supernatants to the culture medium of monocytes, and we then cultured those monocytes under either normoxic or hypoxic conditions with GM-CSF (or M-CSF) for 7 days before analyzing their morphology. We found that a mixture of round and spindle-like macrophages formed, and the percentage of spindle-like macrophages and CD163+ cells were altered by differences in the type of hypoxic stimulation that was applied. The results were more robust in the GM-CSF group, and these results are reflected in the images shown in Figure 4. These results imply that both the hypoxic environment and hypoxia-treated glioma cell supernatants were able to increase the proportion of spindle-like macrophages and the proportion of CD163+ cells (Figure 4B-4F, 4H, 4I). The macrophages that were induced by both hypoxia and the hypoxia-treated glioma cell supernatants contained the highest proportions of spindle-like and CD163+ cells (Figure 4E, 4F, 4H, 4I), imply the presence of a synergetic effect between these two hypoxic conditions on the M2 polarization of TAMs.

### The influence of hypoxia and hypoxia-stimulated glioma cell culture supernatants on cytokine secretion and gene expression in HMDMs

**Figure 5:**
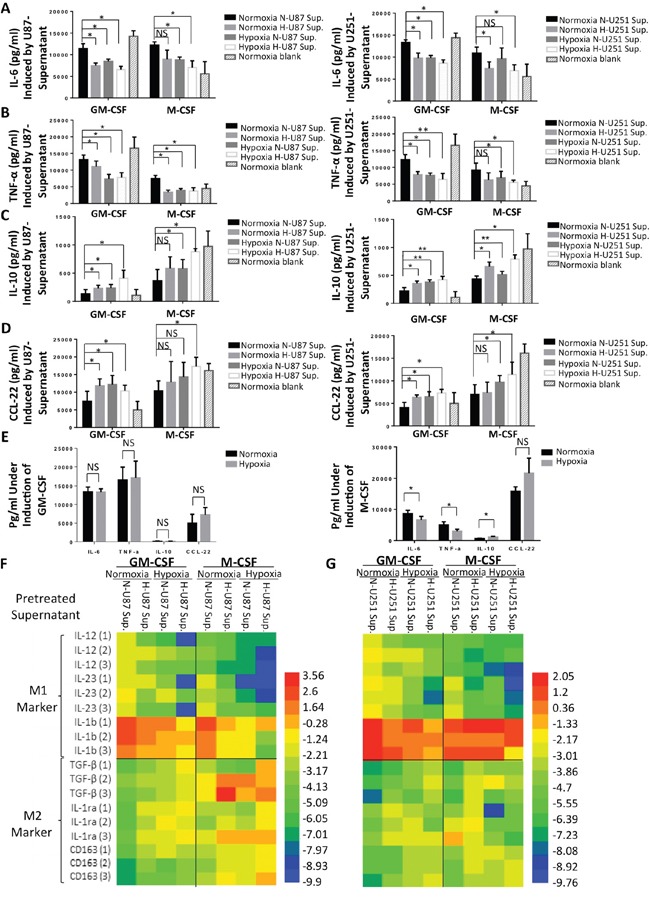
Cytokine secretion and gene expression by HMDMs after hypoxic stimulations **A-D.** Human monocytes were stimulated as described in Figure 4B and 4C. On day 7, the macrophages were stimulated for 24 h using LPS+IFN-γ. After 24 h, the concentrations of the cytokines IL-6, TNF-α, IL-10 and CCL-22 were determined in the HMDM culture supernatants. The same procedures was followed for human monocytes that were cultured with U251 cell supernatants. **E.** Human monocytes were cultured for 7 days with only GM-CSF or M-CSF under normoxic or hypoxic conditions. The cells were then stimulated for another 24 h using LPS and IFN-γ, and the supernatants of the macrophages were subsequently collected to analyze cytokine levels. **F** and **G.** Heatmap showing the gene expression of IL-12, IL-23, IL-1b, TGF-β, IL-1ra and CD163 in HMDMs after the cells were stimulated using same conditions described above.

We next studied cytokine profiles of HMDMs, which are major characteristics that distinguish different macrophage functional phenotypes. Human monocytes were collected and stimulated for 7 days using different hypoxic conditions, as described in Figure 4 (normoxic/hypoxic environment + normoxia-/hypoxia-treated glioma cell supernatants) in the presence of GM-CSF or M-CSF. On day 7, the HMDMs were stimulated using LPS and IFN-γ for another 24 h. Then, the supernatants were collected from HMDMs, and the concentrations of the cytokines IL-6, TNF-α, IL-10 and CCL-22 were determined. None of these cytokines was observed in either U87 or U251 cell supernatants. These results suggested that in macrophages, exposure to either a hypoxic environment (+ normoxia-treated glioma supernatants) or hypoxia-treated glioma supernatants (+ normoxia) resulted lower levels of expression of M1 markers (e.g., IL-6 and TNF-α) and higher levels of M2 marker expression (e.g., IL-10 and CCL-22) than were observed in the control group (normoxia + normoxia-treated glioma supernatants) (Figure 5A-5D). The macrophages that were stimulated using both a hypoxic environment and hypoxia-treated glioma cell supernatants expressed even lower levels of M1 marker cytokines and higher levels of M2 marker cytokines than the macrophages that were exposed to only one hypoxic factor (i.e., either a hypoxic environment or hypoxia-treated glioma supernatants). However, the HMDMs that were induced using GM-CSF were more sensitive to stimulation with hypoxia than the M-CSF-induced macrophages. CCL-22 secretion showed the lowest level of conformity with the trend toward M2 polarization that was observed in the M-CSF groups. To determine whether the hypoxic environment itself, without the tumor supernatants, was capable of driving macrophages toward the alternative phenotype, we cultured monocytes with one of either GM-CSF or M-CSF under normoxic or hypoxic conditions. We found that the macrophages that were cultured with GM-CSF did not demonstrate hypoxia-inducible changes in cytokine secretion (Figure 5E, left), whereas the macrophages that were induced using M-CSF showed more features of M2 TAMs (Figure 5E, right). Because the level of M-CSF in the culture medium was constant while the cells were stimulated with hypoxia, we predicted that the hypoxia itself may have increase M-CSFR in the macrophages, and that this, in turn, promoted M2 polarization. After the supernatants of the HMDMs were collected, the genetic profiles of the cells were detected. We compared the expression of 6 genes, including the M1 markers IL-12, IL-23, and IL-1b (M1 markers) and the M2 markers TGF-β, IL-1ra and CD163 between different experimental groups. The changes we observed in gene levels were similar to the changes we observed in the secretion of cytokines: both a hypoxic environment and hypoxia-treated glioma cell supernatants had an M2-polarizing effect on macrophage gene expression. The most obvious changes in the genetic profiles of the cells that indicated the activation of the alternative macrophage phenotype were observed in the hypoxia + hypoxia-treated glioma supernatant group (Figure 5F-5H). The details of the data from these experiments are shown in Supplementary Figure S5.

### HIF signaling is inhibited by treatment with ACF in glioma cells and macrophages

**Figure 6:**
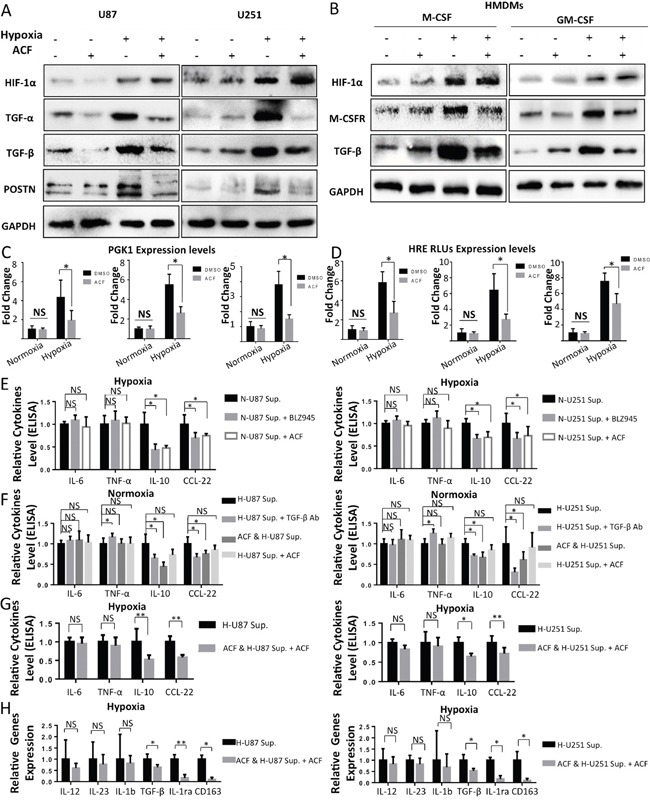
ACF partially reverses M2 macrophage polarization in a HIF-dependent manner **A.** Western blot analysis of HIF-1α, TGF-α, TGF-β, and POSTN levels in U87 and U251 cells that were cultured under normoxic or hypoxic conditions for 2 days with or without ACF. **B.** Human monocytes were cultured in the presence of GM-CSF or M-CSF for 7 days under normoxic or hypoxic conditions and with or without ACF. The HMDMs were then stimulated for 24 h using LPS + IFN-γ. Western blot analysis showing HIF-1α, M-CSFR, and TGF-β levels in the cells shown above. **C.** The gene expression level of the HIF target Pgk-1 was analyzed in U87/U251 cells and THP-1 macrophage-like cells that were grown under normoxic/hypoxic conditions in the presence of PBS or ACF. **D.** THP-1-induced macrophages and U87/U251 cells were transfected with a plasmid containing firefly luciferase under the control of the VEGF promoter (with three consecutive HRE sequences) and cultured under normoxic or hypoxic conditions in the presence or absence of ACF. *, P <0.05, **, P <0.01, NS, P>0.05 (n=3, mean ± s.e.m., two-tailed unpaired t test). **E.** ACF (3 μM) or BLZ945 (670 nM) or DMSO (1 μL/ml) was added to the culture medium of human monocytes, and the cells were then grown under hypoxic conditions with normoxia-treated glioma supernatants (20%) in the presence of GM-CSF for 7 days. On day 7, the macrophages were stimulated for 24 h with LPS + IFN-γ, and cytokine levels were analyzed in the supernatants. **F.** Human monocytes were cultured for 7 days with GM-CSF under normoxic conditions in the presence of hypoxia-treated glioma cell supernatants (H-U87/251 Sup.), hypoxia-treated glioma cell supernatants + anti-TGF-β antibodies (H-U87/251 Sup. + TGF-β Ab), ACF-pretreated hypoxia-stimulated glioma cell supernatants (ACF & H-U87/251 Sup.) or hypoxia-stimulated glioma cell supernatants + ACF (3 μM) (H-U87/251 Sup. + ACF). The levels of cytokines were then detected in the culture supernatants. **G.** Human monocytes were cultured for 7 days with GM-CSF in the presence of a hypoxic environment + hypoxia-treated glioma cell supernatants or a hypoxic environment + ACF-pretreated hypoxia-stimulated glioma cell supernatants + ACF (at a final concentration of 3 μM). On day 7, the macrophages were stimulated for 24 h using LPS + IFN-γ. Cytokine levels were then determined. **H.** Gene expression levels of IL-12, Il-23, IL-1b, IL-1ra, TGF-β and CD163 in HMDMs that were stimulated as described above in the (G) groups. *, P <0.05, **, P <0.01, NS, P>0.05 (n=3 donors, mean ± s.e.m., two-tailed paired t test).

M-CSFR is a principal receptor for M-CSF on macrophages, and the M-CSFR inhibitor BLZ945 has been reported to reduce M2 TAM enrichment in gliomas [48]. TGF-β is a multifunctional peptide that also has potential functions during M2 polarization in macrophages [49]. Both of these proteins are downstream of HIF. The effect of the hypoxia-induced upregulation of M-CSFR on macrophages and TGF-β levels in glioma cells are likely to be components in the pathway that enables the M2 transformation of TAMs in gliomas. Because it is a potent HIF inhibitor, we were interested to determine whether ACF could block the increased expression of POSTN, M-CSFR and TGF-β and further inhibit the hypoxia-induced recruitment and M2-polarizing effects of TAMs in gliomas. We first attempted to identify the specific HIF subunit that mediates the enhanced expression of POSTN, TGF-β and M-CSFR under hypoxic conditions. As shown in Supplementary Figure S6A, POSTN, TGF-β, and M-CSFR were expressed at higher levels in gliomas and HMDM cells that were exposed to hypoxia than in cells that were cultured under normoxic conditions. Silencing HIF-1α using a specific siRNA significantly but incompletely suppressed the increase in the amplitude of POSTN and TGF-β expression in U87/U251 cells that were treated with hypoxia (Supplementary Figure S6A, S6B). Similarly, the hypoxia-induced enhancement in the expression of TGF-β and M-CSFR was also impaired by an HIF-1α siRNA (Supplementary Figure S6C, S6D) in HMDMs. In addition, we found that the hypoxia-induced up-regulation of these molecules was blocked by pretreatment with ACF in U87 and U251 cells and HMDMs (Figure 6A, 6B). ACF had no direct effect on the expression of HIF-1α in either glioma cells or HMDMs, a finding that is in agreement with the results described in other reports [34]. To further test the impact of ACF on HIFs, we sought to determine whether ACF alters the expression of the HIF target phosphoglycerate kinase 1 (Pgk1) at the transcriptional level. We found that Pgk1 levels were markedly reduced at the transcriptional level in both glioma cells and HMDMs when they were pretreated with ACF before they were stimulated with hypoxia (Figure 6C). In addition, administering ACF decreased HRE-driven luciferase reporter gene expression in both glioma cells and THP-1 macrophage-like cells exposed to hypoxic conditions (Figure 6D).

### ACF partially reverses hypoxia-induced M2 polarization in macrophages

ACF may perform its anti-M2 polarization function in 2 ways. It inhibits the hypoxia-induced upregulation of M-CSFR expression in macrophages and the production of TGF-β in both glioma cells and macrophages. Here, we used GM-CSF rather than M-CSF to induce macrophages because we used an M-CSFR inhibitor (BLZ945) in these experiments, and the presence of M-CSF might therefore have altered the results. We first added ACF (3 μM) to the culture medium of human monocytes while the cells were being grown in a hypoxic environment (20% normoxia-treated glioma cell medium). BLZ945, a potent M-CSFR inhibitor, was used in a control group to determine whether the anti-M2 polarizing function of ACF is mediated by the suppression of M-CSFR. We found that both ACF and BLZ945 decreased the hypoxia-induced upregulation of the secretion of two M2 TAM markers: the cytokines IL-10 and CCL-22. However, they were not able to reverse the reduction in the expression of the M1 markers IL-6 and TNF-α (Figure 6E). To determine the impact of ACF on hypoxia-treated glioma supernatants, we next added ACF to the culture media of U87 and U251 cells and then incubated the cells in a hypoxic environment for 2 days to obtain ACF-pretreated hypoxia-stimulated U87 and U251 cell supernatants. These supernatants were then used in co-cultures (20%) with human monocytes. In these experiments, we sought to determine whether ACF could change the direction of glioma TAM polarization by exerting an influence on glioma cell cytokine secretion. In a control group, an anti-TGF-β neutralizing antibody was added to the hypoxia-treated glioma supernatants to determine whether ACF impairs glioma TAM M2 polarization by decreasing the hypoxia-induced secretion of TGF-β from glioma cells. IL-10 and CCL-22 were secreted from macrophages at lower levels when the cells were exposed to ACF-pretreated hypoxia-stimulated glioma supernatants than from cells exposed to non-ACF-pretreated hypoxia-stimulated-U87/U251 supernatants. The anti-TGF-β antibody group showed similar results, except that the secretion of TNF-α was higher in the U251 group (Figure 6F). Finally, we analyzed the function of ACF in macrophage polarization by exposing cells to a combination of two hypoxic conditions (hypoxia + hypoxia-treated glioma supernatants). ACF also reduced M2 marker expression in macrophages in this group (Figure 6G, 6H). Collectively, these results indicate that treatment with ACF decreased the expression of M2 TAM marker expression and cytokine production in HMDMs that were exposed to hypoxic conditions, but that ACF did not reverse the reduction of M1 TAM markers.

### ACF-treated tumors exhibit decreased sizes and macrophage infiltration

**Figure 7:**
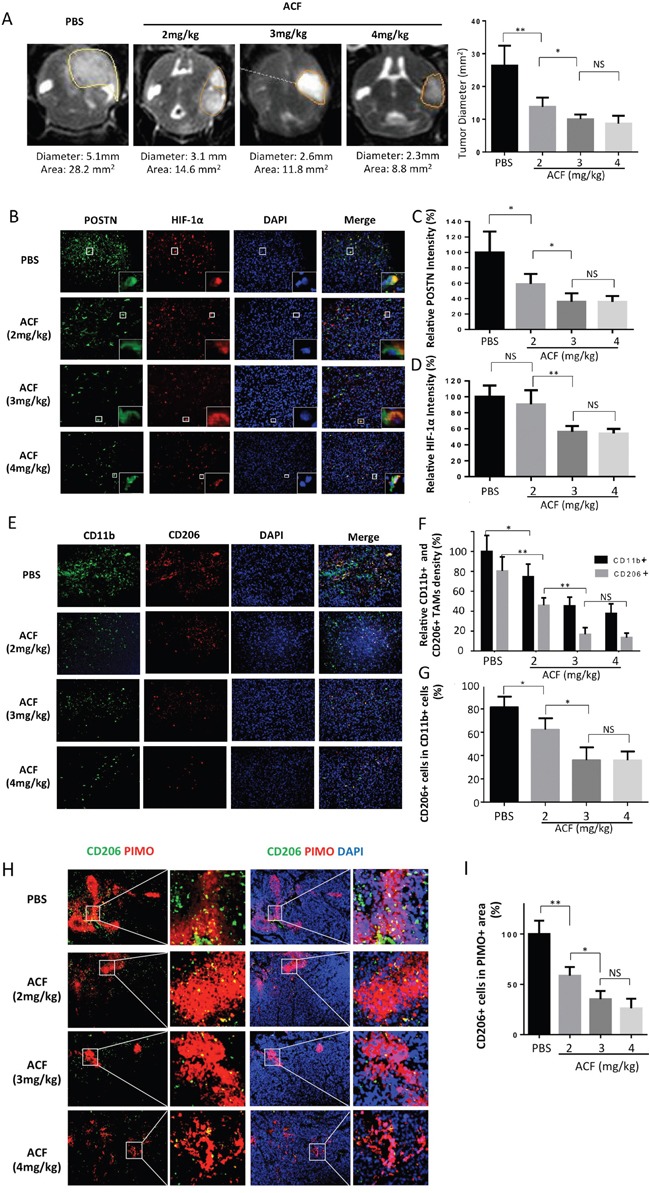
ACF treatment limits glioma progression and M2 type TAM infiltration **A.** Representative images of T2-weighted MRI scans of animals in the PBS- or ACF-treated groups at 6 weeks post-injection (*in situ*) of U87 cells. The line indicates the region of interest that was used to calculate the tumor volume. A graphical analysis of the tumor diameters, as observed in MRI images of the different glioma model groups. *, P <0.05; *, P <0.01; (n=5 tumors, mean ± s.e.m., two-tailed unpaired t test). **B.** Representative immunofluorescence images of *in situ* glioma sections that were obtained from animals in different groups of mice and then stained for POSTN (green), HIF-1α (red) and DAPI (blue). **C** and **D.** Graphical analysis of POSTN and HIF-1α expression in gliomas that were obtained from animals in the PBS- and ACF-treated groups showing that both POSTN and HIF-1α expression were decreased by treatment with ACF. **E.** Representative immunofluorescence images of *in situ* glioma sections that were obtained from different groups of mice and stained for the TAM marker CD11b (green), the M2 macrophage marker CD206 (red) and DAPI (blue). **F** and **G.** Graphical analysis of CD11b and CD206 showing that both TAM infiltration and the proportion of M2 type TAMs were lower when the mice were treated with ACF. **H.** Representative image showing co-localization between CD206 immunofluorescence and pimonidazole (PIMO) staining in the tumor. Scale bars: 200 μm. **I.** Graphical analysis of (H) showing that there was a decrease in M2 TAM infiltration in hypoxic areas after treatment with ACF. *, P <0.05, **, P <0.01, NS, P >0.05 (n=5 tumors, mean ± s.e.m., one-way ANOVA test).

We next examined the anti-tumor potential of ACF in a glioma mouse model. Mice received daily i.p. injections of PBS or ACF (2 mg/kg/d, 3 mg/kg/d or 4 mg/kg/d) for 4 weeks. T2WI-MRI was used to assess tumor volume. The cumulative data from 5 independent experiments showed that in the 6^th^ week, the average tumor size was clearly lower in the ACF-injected mice than in the control mice (PBS). Tumor size in the 3 mg/kg group was smaller than tumor size in the 2 mg/kg group, but there was no significant difference in tumor size between the 4 mg/kg group and the 3 mg/kg group (Figure 7A). However, the difference in tumor size between the 3 groups that were administered different doses of ACF were not prominent at the third week after implantation. (Supplementary Figure S7). We then performed immunofluorescence staining to analyze protein expression and TAM infiltration in tumor tissues. These experiments demonstrated that infiltration by total TAMs (CD11b+ cells) and M2 type TAMs (CD206+ cells) were both ablated in the ACF groups. However, no significant difference was observed between the 4 mg/kg and 3 mg/kg groups (Figure 7B). HIF-1α expression was not significantly different between the 2 mg/kg group and the control group, but it was lower in the 3 mg/kg and 4 mg/kg groups (Figure 7C, 7D). POSTN expression was decreased in all ACF groups, but no difference was observed between the 3 mg/kg and 4 mg/kg groups (Figure 7E-7G). Moreover, we performed a co-immunofluorescence analysis of PIMO (a hypoxyprobe that reveals the hypoxic region in tissue) and CD206 (a M2 TAM marker). We found that the M2 type macrophages (CD206+) were highly enriched in hypoxic regions (PIMO+) in glioma xenografts (Figure 7H). In the ACF 2 mg/kg group, the hypoxic areas were not significantly reduced. However, infiltration by M2 TAMs into hypoxic areas was significantly attenuated. The density of CD206+ macrophages continued to decrease in the ACF 3 mg/kg group (Figure 7H). No difference was observed in the density of M2 TAM infiltration into the hypoxic region between the ACF 3 mg/kg and 4 mg/kg groups (Figure 7H, 7I). IHC staining for M-CSFR, TGF-β and HIF-1α also showed that these markers were decreased at the protein level after treatment with ACF (Supplementary Figure S8). These data demonstrate that TAMs are enriched in hypoxic regions in gliomas and that administering ACF could reduce the enrichment of hypoxia-induced M2 TAMs *in vivo*.

## DISCUSSION

**Figure 8:**
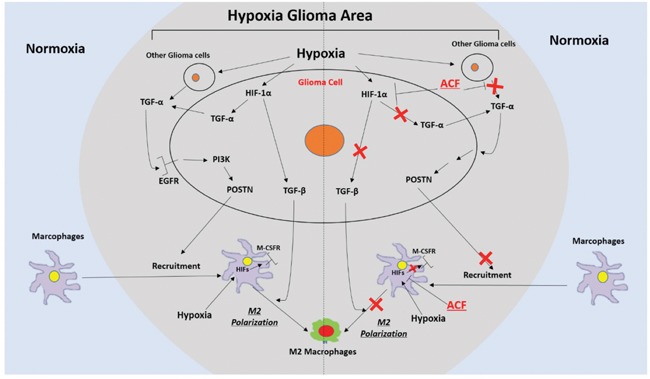
Schematic representation of the recruitment of TAMs and their M2 polarization in hypoxic glioma areas and a description of a mechanism by which ACF may alter these two processes

TAMs have emerged as potential targets for anticancer therapies. However, to translate TAM-targeted therapies into therapeutic practice, we need to obtain a better understanding of the mechanisms that drive the recruitment and polarization of TAMs. Hypoxia-responsive HIF proteins play essential roles in promoting M2 TAM infiltration via multiple mechanisms. ACF, a classic HIF inhibitor, has already been shown to be safe and to produce only rare side effects in patients when used for up to 5 months [50]. It was therefore selected as a potential TAM-targeted anti-tumor drug for our experiments. In this study, we demonstrated that hypoxia enhanced the recruitment of TAMs by upregulating POSTN expression in glioma cells. TAMs were localized close to perivascular niches in low-HIF-1α glioma tissue and their distribution became more disseminated as HIF-1α positive regions increased. The hypoxic glioma microenvironment polarized TAMs toward the M2 subtype by increasing the expression of M-CSFR in macrophages and TGF-β in glioma cells. Moreover, ACF reduced glioma progression *in vivo* and inhibited the recruitment and M2 polarization of TAMs (Figure 8).

The enhanced directional migration of macrophages toward hypoxic areas has been attributed to the hypoxia-inducible expression of POSTN in glioma cells. Interestingly, macrophage migration was impaired when cells were exposed to hypoxia (Figure 2V, 2O). This phenomenon may partially explain the mechanism by which macrophages become trapped in hypoxic regions after they were initially attracted to them.

Hypoxia, TAMs and GSLCs have all been observed in GSLC niches in gliomas [39, 45]. We found that in low HIF-1α-expressing GBMs, POSTN was expressed primarily around CD31+ vessels. Two chemotactic molecules, SDF-1α and OPN, were also found to be expressed in and around these perivascular niches. The congregation of these macrophage chemotactic factors in perivascular niche areas may partially explain the accumulation of TAMs around vessels in low HIF-1α glioma specimens. Because hypoxia and TAMs play supportive roles in the survival and maintenance of tumor stem cells [51, 52], the enrichment of TAMs in perivascular niches may contribute to the propagation of GSLCs. As HIF-1α positive regions expanded, more non-glioma stem-like cells began to express POSTN (Supplementary Figure S3G, S3J). We found that the expression level and range of POSTN were each much higher and more disseminated in high-HIF-1α-expressing glioma sections than in low-HIF-1α expressing glioma specimens. While SDF-1α and OPN were also slightly increased in perivascular areas in high-HIF-1α glioma tissue, their expression levels and areas were much smaller than those of POSTN (Supplementary Figure S3K-S3V). TAMs are therefore attracted to expanded hypoxic areas by POSTN.

Because M2 TAMs congregate in hypoxic areas in gliomas [53, 54], we originally predicted that hypoxia would directly drive the acquisition of the M2 phenotype in macrophages. However the TAMs in colon cancer, which also presents large hypoxic areas [55], are mainly M1 type [56]. In addition, when we cultured human monocytes under hypoxic conditions in the presence of GM-CSF, no TAM re-specification was observed. However, when human monocytes were exposed to a combination of hypoxia and M-CSF, they were induced to undergo a stronger M2 polarization (Figure 4A, 4G). Based on these findings, we determined that hypoxia alone is not enough to promote TAM M2 polarization. The tumor microenvironment, which includes cytokines and other cell components, also plays essential roles in hypoxia-induced TAM polarization. Consequently, we investigated the effect of “hypoxia” on macrophages using two different experimental paradigms: a physically hypoxic environment and an environment that was induced using hypoxia-treated glioma cell supernatants (it should be noted that here, “a physically hypoxic environment” means macrophages that were exposed to hypoxia via the presence of glioma cell supernatants and not hypoxia alone). Although the effect of hypoxia on macrophage subtype switching has been studied [57, 58], no previous study has distinguished between the separate roles played by these two hypoxic components in macrophage polarization. Our results show that both of these components of the hypoxic environment promoted M2 polarization in TAMs in most of the experimental groups.

Many approaches have been used to ablate TAMs or inhibit their tumor-promoting functions in mouse models of cancer [59]. Our study shows that blocking the hypoxia-inducible expression of M-CSFR in TAMs and TGF-β in glioma cells using ACF or a specific inhibitor (e.g., BLZ-945 or anti-TGF-β antibodies) impaired the hypoxia-inducible M2 polarization of TAMs. An anti-TGF-β antibody increased the secretion of TNF-α from HMDMs that were exposed to hypoxia-treated glioma cell supernatants, but ACF did not reverse the expression of M1 TAM cytokines, implying that the anti-M2 effect of ACF is only partly mediated by the inhibition of the hypoxia-inducible production of TGF-β. Because M-CSFR is also involved in supporting the survival, extravasation and recruitment of TAMs [60, 61], the impact of suppressing the expression of M-CSFR on TAMs involves more than an anti-M2 polarizing effect. Mice treated with ACF consistently developed smaller glioma tumors that contained markedly lower numbers of recruited TAMs. We found that the differences between 3 mg/kg/d group and 4 mg/kg/d group in tumor size, the expression of POSTN, M-CSFR, and TGF-β and the infiltration of TAMs into hypoxic areas were not significant. These data implied that 3 mg/kg/d was the optimal dose that produced the maximum pharmacological effect in this glioma mouse model. No difference was observed in HIF-1α expression between the 2 mg/kg/d group and the control group. These results might be explained by the fact that in the 2 mg/kg/d group, glioma size was bigger than 3 mm and that the hypoxic conditions in the tumor were not improved. ACF did not alter the production of HIF-1α but did decrease its dimerization, which did not alter HIF-1α expression on an immunofluorescence staining image. However, as tumor size decreased, the oxygen supply became abundant within the tumor region, and this in turn reduced the expression of HIF-1α. As a result, the expression of HIF-1α was decreased in the 3 mg/kg/d and 4 mg/kg/d groups. We have therefore shown that macrophage polarization and infiltration into the tumor microenvironment progresses in an HIF-dependent manner under hypoxic conditions. It is becoming increasingly clear that HIFs form a link between hypoxia and tumorigenesis through their activity in macrophages during cancer progression.

Our studies have shed light on the mechanisms that underlie TAM recruitment and M2 polarization under hypoxic conditions in gliomas. We have also shown that the HIF inhibitor ACF may exert a significant suppressive effect on glioma progression by inhibiting TAM enrichment and alternative activation. However, the CNS immune microenvironment is a highly complex system, and focusing so strongly on HMDMs prevents researchers from studying the whole tumor immune microenvironment. Other immune cells, such as MDSC and lymphocytes, may also play critical roles in tumor progression. Moreover, culturing immune cells with tumor cell supernatants only partially mimics the tumor microenvironment because there cell-to-cell contact-mediated communication cannot be tested under these conditions. More importantly, the mechanism by which the macrophage cytoskeleton is altered in the glioma hypoxic environment, which impacts macrophage migration, was not studied in our experiments. In the future, we will continue our investigations into these areas. Finally, obtaining a better understanding of the effects of hypoxia on TAMs and the inhibition of HIFs in both glioma cells and recruited macrophages will provide us with new insights with which to investigate the relationships between hypoxia and cancers.

## MATERIALS AND METHODS

### Tissue samples, monocytes and cell lines

The human acute monocytic leukemia cell lines THP-1 and the human glioma cell lines U87 and U251 were purchased from the Chinese Academy of Sciences Cell Bank. Forty-two human glioma tissues and five normal brain tissues were obtained from the Department of Neurosurgery of Qilu Hospital of Shandong University. All glioma specimens were classified by two experienced clinical pathologists according to the WHO Classification of Tumors guidelines. PBMCs were isolated from the blood of healthy volunteers according to the guidelines 2005/61/EG, 2004/33/EG, 2002/98/EG and 2005/62/EG of the EU and the Helsinki Declaration. All donors provided informed consent. Blood was separated using standard density gradient centrifugation (30 minutes at 2000 rpm at 21°C, LTS1077; Tina Jin Hao Yang Bio.). PBMCs were extracted from the interphase. PBMC fractions were incubated with magnetic CD14-positive beads (Miltenyi Biotec, 103-050-201), and CD14-positive monocytes were trapped using a magnet and an LS-positive selection column (Miltenyi Biotec, 130-042-401). Monocytes were extracted from the column after the column was washed. The monocytes were washed in complete medium. PBMCs were resuspended in complete culture medium, counted, and plated at 5 × 10^6^ cells/well in 6-well flat-bottom plates [56]. Our study was approved by the Institutional Review Board of Shandong University. Written informed consent was obtained from all participants, and the hospital ethical committee approved the experiments.

### Reagents and cell culture

U87 and U251 cells were cultured in DMEM (10% FBS), and THP-1 cells were cultured in RPMI-1640 (10% FBS). All cells were maintained in 20% O_2_ at 37°C in a 5% CO_2_ humidified chamber. Hypoxic conditions were induced by incubating the cells under 1% O_2_, 5% CO_2_, and 94% N_2_ at 37°C. PD153035 (BioVision), LY294002 (Beyotime Biotechnology), BLZ945 (selleck), TGF-α (PeproTech) and Recombinant POSTN (Biovision) were purchased.

### Cell transfection

The POSTN siRNA (POSTN-homo-1205 for 1205, POSTN-homo-876 for 876) and scrambled inhibitor control were designed and synthesized by GenePharma (China). HIF-1α siRNA (sc-35561) was bought from Santa Cruz Biotechnology. Cell transfections were performed using Lipofectamine-2000 according to the manufacturer's protocols. Cells transfected with scrambled oligos were used as negative controls.

### Induction of human M1/M2 monocyte-derived macrophages

The PBMCs were cultured for 7 days in complete 1640 culture medium containing 10% FBS and supplemented with either 50 ng/mL recombinant human GM-CSF to generate M1 macrophages or 50 ng/mL recombinant human M-CSF to generate M2 macrophages. A 1.5 mL volume of medium was replaced with 1.5 mL of fresh medium containing GM-CSF (215-GM, R&D Systems) or M-CSF (216-MC, R&D Systems) on day 4. On day 7, the macrophages were harvested and seeded in duplicate wells at 1 × 10^5^ cells/mL and 200 μL/well in 96-well flat-bottom culture plates and then stimulated for 24 h using LPS (Solarbio, L8880) (1 μg/mL) or IFN-γ (PeproTech, 300-02) (20 ng/mL). Culture supernatants were collected after 24 h and stored at -80°C until use [11, 62].

### Production of conditioned supernatant

To produce conditioned medium, glioma cells were seeded 5 × 10^6^ cells/well in 6-well flat-bottom plates and allowed to adhere overnight. They were then extensively washed and incubated for 48 h with fresh complete DMEM containing different stimulants (e.g., with or without 4 μM ACF) or under different environmental oxygen conditions (e.g., normoxia, 20% O_2_ or hypoxia, 1% O_2_). Conditioned media was collected, centrifuged at 5000 ×g, filtered using a 0.2-μm filter (Millipore) and stored at -80°C until use.

### IHC and immunofluorescence staining

IHC staining was performed using an ABC kit (Origene) and a DAB kit (Zhongshanjinqiao) according to the manufacturer's instructions. Primary antibodies against POSTN (1:300/1:200), HIF-1α (ab16066, 1:600/1:100), CD11b (ab133357, 1:800/1:200), CD206 (ab64693, ab8918 1:100/1:50), M-CSFR (ab183316 1:100/no), TGF-β (ab27969, 1:500/no), SDF-1 (ab9797 no/1:100), osteopontin (ab91655 no/1:100), CD31 (ab24590, ab76533 no/1:100), CX3CR1 (ab8021, no/1:50) and CCR2 (ab176390, no/1:50) were purchased from Abcam. PIMO hypoxyprobe 1MAB1 (Hypoxyprobe Incorporation, USA) and CD133 (Miltenyi Biotec) were used for IHC and immunofluorescence staining.

### Semi-quantitative estimations of immunostaining

Sections were evaluated in a blinded manner by two experienced investigators who provided a consensus opinion of staining patterns that were observed under light microscopy. Each of the 3 tissue cores from each patient tumor sample was evaluated, and the mean of the results was determined. The POSTN and HIF-1α immunostaining scores were estimated using both the percentage of positively stained tumor cells and the staining intensity. The percentage of positivity was scored as “0” (<5%, negative), “1” (5–25%, sporadic), “2” (25–50%, focal), or “3” (>50%, diffuse). The staining intensity was scored as “0” (no staining), “1” (weakly stained), “2” (moderately stained), or “3” (strongly stained). Both the percentage of positive cells and the staining intensity were evaluated under double-blind conditions. The immunostaining scores were calculated as the percentage positive score multiplied by the staining intensity score, and they ranged from 0 to 9. The glioma patients were divided into two groups based on immunostaining scores: a low expression group (score ≤ 3) and a high expression group (score > 3).

To evaluate the proportions of positively stained CD11b and CD206 cells, the 5 high-power magnification fields (400×) with the most abundant distributions of positive cells were selected from each specimen. The positively stained and unstained cells were then counted in these regions. The percentage of positively stained cells was recorded, and the glioma patients were divided into two groups according to the results: a low expression group (≤25%) and a high expression group (>25%).

### RNA extraction and real-time quantitative PCR

Total RNA was extracted using TRIzol Reagent (Takara), and cDNA was synthesized using a ReverTra Ace qPCR RT Kit. Relative gene expression levels were determined using SYBR Premix Ex TaqTM Kit and the primers shown in Supplementary Table S2. The comparative threshold cycle method was used to calculate gene expression levels, and the results were normalized to GAPDH, which was used as the gene reference.

### Migration and Invasion assays

Cell migration assays were performed using Transwell chambers measuring 6.5 mm in diameter (8-μm pore size, Corning). In total, 1 × 10^5^ THP-1 induced macrophages in FBS-free medium were seeded in the upper chambers of uncoated Transwell chambers. Tumor culture medium was added to the lower chamber. After 12 h, the cells that had migrated to the lower surface were fixed, stained with eosin and counted under a microscope. Five random views were used to count the cells.

### Western blot analysis

Protein extractions from tumor cells or macrophages were analyzed. The following primary antibodies were used: HIF-1α (1:1000, Abcam-ab16066), Periostin (1:1000, Abcam-ab79946), TGF-α (1:200, SANTA CRUZ, sc-9043), TGF-β (1:2000, abcam-ab27969) and M-CSFR (1:1000, Abcam-ab182582).

### Luciferase assay

U87, U251 and THP-1 cells were transfected with plasmids containing firefly luciferase under the control of a wild-type (WT) HRE promoter of the human VEGF gene or a mutant HRE promoter along with a renilla control. At 8 h after transfection, the media were changed and either PBS or ACF was added. The cells were then placed under normoxic or hypoxic conditions for 48 h. Luciferase activity was read using a luminometer (Berthold Mithras-LB940) at 48 h after the addition of PBS or ACF. The Firefly activity in the WT HRE plasmid was normalized to the activity of renilla and the mutant HRE activity.

### Cytokine profiles of HMDMs

To assess M1/M2 polarization in HMDMs, we determined the concentrations of IL-6, TNF-α, IL-10 and CCL-22 in culture supernatants using ELISA kits (Quantikine ELISA kits D6050, DTA00C, D1000B, DMD00 from R&D Systems Inc.).

### Flow cytometry

To detect CD163-positive M2-macrophges, HMDMs were digested and then washed twice with PBS. The macrophages were then harvested for flow cytometry. The cells were suspended in PBS and incubated with FITC-conjugated anti-CD163 antibodies (eBioscience) for 1 h at room temperature. Isotype controls were run in parallel. The cells were analyzed using flow cytometry with a BD Accuri C6 flow cytometer (BD Biosciences, USA).

### Orthotopically xenografted glioma mouse model

Intracranial brain tumor xenografts were obtained by stereotactically implanting U87 cells (1×106 cells per mouse) into the brains of five-week-old male BALB/c nude mice (Chinese Academy of Sciences). For all *in vivo* experiments, the mice were administered ACF (Sigma, M.W. 259.7) from 2 weeks after implantation for 4 weeks via daily i.p. injections (2 mg/kg, 3 mg/kg or 4 mg/kg dissolved in PBS) or an equivalent volume of PBS alone. The animals were imaged using T2-weighted MRI (T2WI-MRI) every 20 days. On day 42, the mice were intraperitoneally injected (80 mg/kg) with pimonidazole (Hypoxyprobe Incorporation, USA) 2 h before they were sacrificed, in accordance with ethical guidelines. After the mice were sacrificed, their whole brains were removed and fixed in 4% paraformaldehyde.

### Statistical analysis

All experiments were performed three times. The statistical analyses were performed and experimental graphs were generated using SPSS 17.0 and GraphPad Prism software, respectively. Descriptive statistics, including the mean ± s.e.m. and paired/unpaired Student's t-test, Kaplan-Meier plots, the log-rank test, and one-way ANOVA tests, were used to analyze the significance of differences. P values of less than 0.05 were considered significant. *p < 0.05, **p < 0.01, and ***p < 0.001; NS P>0.05.

## SUPPLEMENTARY MATERIALS FIGURES AND TABLES



## References

[1] Ohgaki H (2009). Epidemiology of brain tumors. Methods Mol Biol.

[2] Ohgaki H, Kleihues P (2005). Epidemiology and etiology of gliomas. Acta Neuropathol.

[3] Charles NA, Holland EC, Gilbertson R, Glass R, Kettenmann H (2012). The brain tumor microenvironment. Glia.

[4] Gordon S, Taylor PR (2005). Monocyte and macrophage heterogeneity. Nat Rev Immunol.

[5] Chang CI, Liao JC, Kuo L (2001). Macrophage arginase promotes tumor cell growth and suppresses nitric oxide-mediated tumor cytotoxicity. Cancer Res.

[6] Mantovani A, Sozzani S, Locati M, Allavena P, Sica A (2002). Macrophage polarization: tumor-associated macrophages as a paradigm for polarized M2 mononuclear phagocytes. Trends Immunol.

[7] Mantovani A, Sica A, Sozzani S, Allavena P, Vecchi A, Locati M (2004). The chemokine system in diverse forms of macrophage activation and polarization. Trends Immunol.

[8] Franklin RA, Liao W, Sarkar A, Kim MV, Bivona MR, Liu K, Pamer EG, Li MO (2014). The cellular and molecular origin of tumor-associated macrophages. Science.

[9] Pucci F, Venneri MA, Biziato D, Nonis A, Moi D, Sica A, Di Serio C, Naldini L, De Palma M (2009). A distinguishing gene signature shared by tumor-infiltrating Tie2-expressing monocytes, blood “resident” monocytes, and embryonic macrophages suggests common functions and developmental relationships. Blood.

[10] Quatromoni JG, Eruslanov E (2012). Tumor-associated macrophages: function, phenotype, and link to prognosis in human lung cancer. Am J Transl Res.

[11] Caras I, Tucureanu C, Lerescu L, Pitica R, Melinceanu L, Neagu S, Salageanu A (2011). Influence of tumor cell culture supernatants on macrophage functional polarization: in vitro models of macrophage-tumor environment interaction. Tumori.

[12] Ling EA, Wong WC (1993). The origin and nature of ramified and amoeboid microglia: a historical review and current concepts. Glia.

[13] Hussain SF, Yang D, Suki D, Aldape K, Grimm E, Heimberger AB (2006). The role of human glioma-infiltrating microglia/macrophages in mediating antitumor immune responses. Neuro Oncol.

[14] Graf MR, Prins RM, Hawkins WT, Merchant RE (2002). Irradiated tumor cell vaccine for treatment of an established glioma. I. Successful treatment with combined radiotherapy and cellular vaccination. Cancer Immunol Immunother.

[15] Pellegatta S, Poliani PL, Corno D, Menghi F, Ghielmetti F, Suarez-Merino B, Caldera V, Nava S, Ravanini M, Facchetti F, Bruzzone MG, Finocchiaro G (2006). Neurospheres enriched in cancer stem-like cells are highly effective in eliciting a dendritic cell-mediated immune response against malignant gliomas. Cancer Res.

[16] Mineharu Y, King GD, Muhammad AK, Bannykh S, Kroeger KM, Liu C, Lowenstein PR, Castro MG (2011). Engineering the brain tumor microenvironment enhances the efficacy of dendritic cell vaccination: implications for clinical trial design. Clin Cancer Res.

[17] Tham M, Khoo K, Yeo KP, Kato M, Prevost-Blondel A, Angeli V, Abastado JP (2015). Macrophage depletion reduces postsurgical tumor recurrence and metastatic growth in a spontaneous murine model of melanoma. Oncotarget.

[18] Chen P, Bonaldo P (2013). Role of macrophage polarization in tumor angiogenesis and vessel normalization: implications for new anticancer therapies. Int Rev Cell Mol Biol.

[19] Bingle L, Brown NJ, Lewis CE (2002). The role of tumour-associated macrophages in tumour progression: implications for new anticancer therapies. J Pathol.

[20] Li Z, Bao S, Wu Q, Wang H, Eyler C, Sathornsumetee S, Shi Q, Cao Y, Lathia J, McLendon RE, Hjelmeland AB, Rich JN (2009). Hypoxia-inducible factors regulate tumorigenic capacity of glioma stem cells. Cancer Cell.

[21] Tripathi C, Tewari BN, Kanchan RK, Baghel KS, Nautiyal N, Shrivastava R, Kaur H, Bhatt ML, Bhadauria S (2014). Macrophages are recruited to hypoxic tumor areas and acquire a pro-angiogenic M2-polarized phenotype via hypoxic cancer cell derived cytokines Oncostatin M and Eotaxin. Oncotarget.

[22] Murdoch C, Lewis CE (2005). Macrophage migration and gene expression in response to tumor hypoxia. Int J Cancer.

[23] Conway SJ, Izuhara K, Kudo Y, Litvin J, Markwald R, Ouyang G, Arron JR, Holweg CT, Kudo A (2014). The role of periostin in tissue remodeling across health and disease. Cell Mol Life Sci.

[24] Bao S, Ouyang G, Bai X, Huang Z, Ma C, Liu M, Shao R, Anderson RM, Rich JN, Wang XF (2004). Periostin potently promotes metastatic growth of colon cancer by augmenting cell survival via the Akt/PKB pathway. Cancer Cell.

[25] Incardona F, Doroudchi MM, Ismail N, Carreno A, Griner E, Anna Lim M (2015). Registered report: Interactions between cancer stem cells and their niche govern metastatic colonization. Elife.

[26] Michaylira CZ, Wong GS, Miller CG, Gutierrez CM, Nakagawa H, Hammond R, Klein-Szanto AJ, Lee JS, Kim SB, Herlyn M, Diehl JA, Gimotty P, Rustgi AK (2010). Periostin, a cell adhesion molecule, facilitates invasion in the tumor microenvironment and annotates a novel tumor-invasive signature in esophageal cancer. Cancer Res.

[27] Zhou W, Ke SQ, Huang Z, Flavahan W, Fang X, Paul J, Wu L, Sloan AE, McLendon RE, Li X, Rich JN, Bao S (2015). Periostin secreted by glioblastoma stem cells recruits M2 tumour-associated macrophages and promotes malignant growth. Nat Cell Biol.

[28] Ouyang G, Liu M, Ruan K, Song G, Mao Y, Bao S (2009). Upregulated expression of periostin by hypoxia in non-small-cell lung cancer cells promotes cell survival via the Akt/PKB pathway. Cancer Lett.

[29] Semenza GL (2007). Life with oxygen. Science.

[30] Jaakkola P, Mole DR, Tian YM, Wilson MI, Gielbert J, Gaskell SJ, von Kriegsheim A, Hebestreit HF, Mukherji M, Schofield CJ, Maxwell PH, Pugh CW, Ratcliffe PJ (2001). Targeting of HIF-α to the von Hippel-Lindau ubiquitylation complex by O2-regulated prolyl hydroxylation. Science.

[31] White JR, Harris RA, Lee SR, Craigon MH, Binley K, Price T, Beard GL, Mundy CR, Naylor S (2004). Genetic amplification of the transcriptional response to hypoxia as a novel means of identifying regulators of angiogenesis. Genomics.

[32] Keith B, Johnson RS, Simon MC (2012). HIF1α and HIF2α: sibling rivalry in hypoxic tumour growth and progression. Nat Rev Cancer.

[33] Imtiyaz HZ, Williams EP, Hickey MM, Patel SA, Durham AC, Yuan LJ, Hammond R, Gimotty PA, Keith B, Simon MC (2010). Hypoxia-inducible factor 2α regulates macrophage function in mouse models of acute and tumor inflammation. J Clin Invest.

[34] Lee K, Zhang H, Qian DZ, Rey S, Liu JO, Semenza GL (2009). Acriflavine inhibits HIF-1 dimerization, tumor growth, and vascularization. Proc Natl Acad Sci U S A.

[35] Shay JE, Imtiyaz HZ, Sivanand S, Durham AC, Skuli N, Hsu S, Mucaj V, Eisinger-Mathason TS, Krock BL, Giannoukos DN, Simon MC (2014). Inhibition of hypoxia-inducible factors limits tumor progression in a mouse model of colorectal cancer. Carcinogenesis.

[36] Wong CC, Zhang H, Gilkes DM, Chen J, Wei H, Chaturvedi P, Hubbi ME, Semenza GL (2012). Inhibitors of hypoxia-inducible factor 1 block breast cancer metastatic niche formation and lung metastasis. J Mol Med.

[37] Wolf Y, Yona S, Kim KW, Jung S (2013). Microglia, seen from the CX(3)CR1 angle. Front Cell Neurosci.

[38] Saederup N, Cardona AE, Croft K, Mizutani M, Cotleur AC, Tsou CL, Ransohoff RM, Charo IF (2010). Selective chemokine receptor usage by central nervous system myeloid cells in CCR2-red fluorescent protein knock-in mice. PLoS One.

[39] Calabrese C, Poppleton H, Kocak M, Hogg TL, Fuller C, Hamner B, Oh EY, Gaber MW, Finklestein D, Allen M, Frank A, Bayazitov IT, Zakharenko SS (2007). A perivascular niche for brain tumor stem cells. Cancer Cell.

[40] Lathia JD, Heddleston JM, Venere M, Rich JN (2011). Deadly teamwork:neural cancer stem cells and the tumor microenvironment. Cell Stem Cell.

[41] Pietras A, Katz AM, Ekstrom EJ, Wee B, Halliday JJ, Pitter KL, Werbeck JL, Amankulor NM, Huse JT, Holland EC (2014). Osteopontin-CD44 signaling in the glioma perivascular niche enhances cancer stem cell phenotypes and promotes aggressive tumor growth. Cell Stem Cell.

[42] Hambardzumyan D, Gutmann DH, Kettenmann H (2016). The role of microglia and macrophages in glioma maintenance and progression. Nat Neurosci.

[43] Ellert-Miklaszewska A, Wisniewski P, Kijewska M, Gajdanowicz P, Pszczolkowska D, Przanowski P, Dabrowski M, Maleszewska M, Kaminska B (2016). Tumour-processed osteopontin and lactadherin drive the protumorigenic reprogramming of microglia and glioma progression. Oncogene.

[44] Hira VV, Ploegmakers KJ, Grevers F, Verbovsek U, Silvestre-Roig C, Aronica E, Tigchelaar W, Turnsek TL, Molenaar RJ, Van Noorden CJ (2015). CD133+ and Nestin+ Glioma Stem-Like Cells Reside Around CD31+ Arterioles in Niches that Express SDF-1α, CXCR4, Osteopontin and Cathepsin K. J Histochem Cytochem.

[45] Heddleston JM, Li Z, McLendon RE, Hjelmeland AB, Rich JN (2009). The hypoxic microenvironment maintains glioblastoma stem cells and promotes reprogramming towards a cancer stem cell phenotype. Cell Cycle.

[46] Ishii A, Kimura T, Sadahiro H, Kawano H, Takubo K, Suzuki M, Ikeda E (2016). Histological Characterization of the Tumorigenic “Peri-Necrotic Niche” Harboring Quiescent Stem-Like Tumor Cells in Glioblastoma. PLoS One.

[47] Li P, Oparil S, Feng W, Chen YF (2004). Hypoxia-responsive growth factors upregulate periostin and osteopontin expression via distinct signaling pathways in rat pulmonary arterial smooth muscle cells. J Appl Physiol.

[48] Pyonteck SM, Akkari L, Schuhmacher AJ, Bowman RL, Sevenich L, Quail DF, Olson OC, Quick ML, Huse JT, Teijeiro V, Setty M, Leslie CS, Oei Y (2013). CSF-1R inhibition alters macrophage polarization and blocks glioma progression. Nat Med.

[49] Sica A, Schioppa T, Mantovani A, Allavena P (2006). Tumour-associated macrophages are a distinct M2 polarised population promoting tumour progression: potential targets of anti-cancer therapy. Eur J Cancer.

[50] Wainwright M (2001). Acridine-a neglected antibacterial chromophore. J Antimicrob Chemother.

[51] Sainz B, Carron E, Vallespinos M, Machado HL (2016). Cancer Stem Cells and Macrophages: Implications in Tumor Biology and Therapeutic Strategies. Mediators Inflamm.

[52] Seidel S, Garvalov BK, Wirta V, von Stechow L, Schanzer A, Meletis K, Wolter M, Sommerlad D, Henze AT, Nister M, Reifenberger G, Lundeberg J, Frisen J (2010). A hypoxic niche regulates glioblastoma stem cells through hypoxia inducible factor 2 α. Brain.

[53] Rampling R, Cruickshank G, Lewis AD, Fitzsimmons SA, Workman P (1994). Direct measurement of pO2 distribution and bioreductive enzymes in human malignant brain tumors. Int J Radiat Oncol Biol Phys.

[54] Prosniak M, Harshyne LA, Andrews DW, Kenyon LC, Bedelbaeva K, Apanasovich TV, Heber-Katz E, Curtis MT, Cotzia P, Hooper DC (2013). Glioma grade is associated with the accumulation and activity of cells bearing M2 monocyte markers. Clin Cancer Res.

[55] Hongo K, Tsuno NH, Kawai K, Sasaki K, Kaneko M, Hiyoshi M, Murono K, Tada N, Nirei T, Sunami E, Takahashi K, Nagawa H, Kitayama J (2013). Hypoxia enhances colon cancer migration and invasion through promotion of epithelial-mesenchymal transition. J Surg Res.

[56] Bogels M, Braster R, Nijland PG, Gul N, van de Luijtgaarden W, Fijneman RJ, Meijer GA, Jimenez CR, Beelen RH, van Egmond M (2012). Carcinoma origin dictates differential skewing of monocyte function. Oncoimmunology.

[57] Fang HY, Hughes R, Murdoch C, Coffelt SB, Biswas SK, Harris AL, Johnson RS, Imityaz HZ, Simon MC, Fredlund E, Greten FR, Rius J, Lewis CE (2009). Hypoxia-inducible factors 1 and 2 are important transcriptional effectors in primary macrophages experiencing hypoxia. Blood.

[58] Burke B, Giannoudis A, Corke KP, Gill D, Wells M, Ziegler-Heitbrock L, Lewis CE (2003). Hypoxia-induced gene expression in human macrophages: implications for ischemic tissues and hypoxia-regulated gene therapy. Am J Pathol.

[59] Ruffell B, Affara NI, Coussens LM (2012). Differential macrophage programming in the tumor microenvironment. Trends Immunol.

[60] Liao J, Feng W, Wang R, Ma S, Wang L, Yang X, Yang F, Lin Y, Ren Q, Zheng G (2016). Diverse in vivo effects of soluble and membrane-bound M-CSF on tumor-associated macrophages in lymphoma xenograft model. Oncotarget.

[61] Van Overmeire E, Stijlemans B, Heymann F, Keirsse J, Morias Y, Elkrim Y, Brys L, Abels C, Lahmar Q, Ergen C, Vereecke L, Tacke F, De Baetselier P (2016). M-CSF and GM-CSF Receptor Signaling Differentially Regulate Monocyte Maturation and Macrophage Polarization in the Tumor Microenvironment. Cancer Res.

[62] Verreck FA, de Boer T, Langenberg DM, Hoeve MA, Kramer M, Vaisberg E, Kastelein R, Kolk A, de Waal-Malefyt R, Ottenhoff TH (2004). Human IL-23-producing type 1 macrophages promote but IL-10-producing type 2 macrophages subvert immunity to (myco)bacteria. Proc Natl Acad Sci U S A.

