# Supplementary material for: Hypoxia promotes glioma-associated macrophage infiltration via periostin and subsequent M2 polarization by upregulating TGF-beta and M-CSFR
Source: Oncotarget. 2016 Sep 2;7(49):80521–42. doi: 10.18632/oncotarget.11825 (PMC5348338; doi:10.18632/oncotarget.11825)
Supplement: Supplementary file 1 [file oncotarget-07-80521-s001.pdf]

Hypoxia promotes glioma-associated macrophage infiltration via periostin and subsequent M2 polarization by upregulating TGF-beta and M-CSFR

SUPPLEMENTARY FIGURES AND TABLES

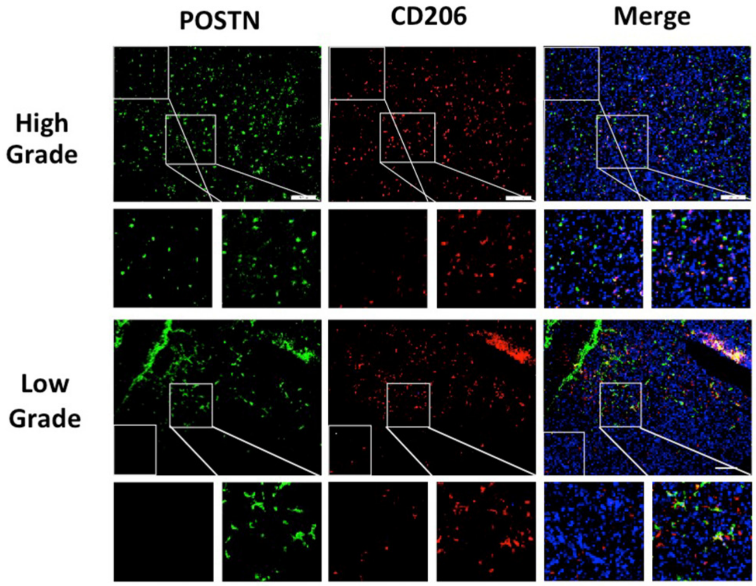

Supplementary Figure S1: Immunofluorescence analysis of POSTN (green) and CD11b (red) expression in glioma tissue slides showing that TAMs are enriched in POSTN-abundant regions. The areas indicated with squares are enlarged and shown under each picture. Scale bars, 200 µm.

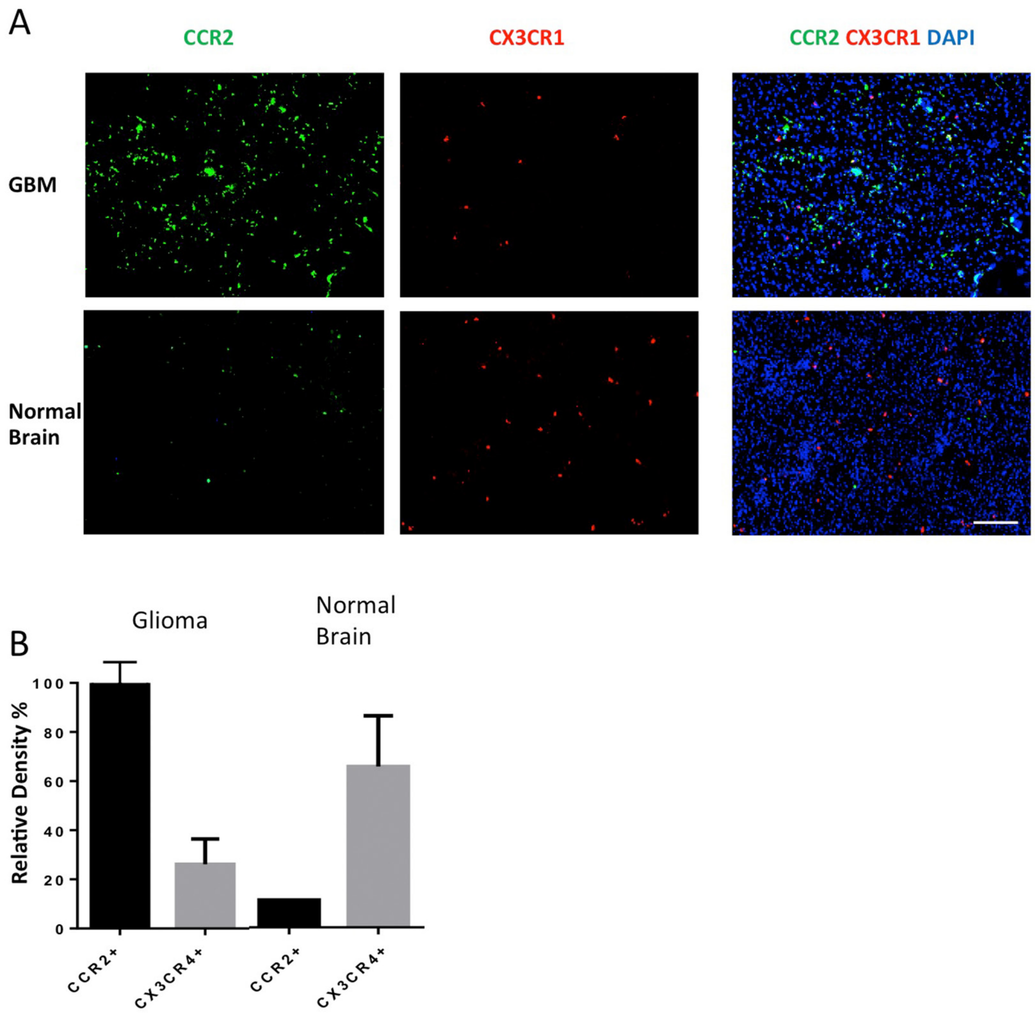

Supplementary Figure S2: TAMs in human gliomas are mainly derived from peripheral blood monocytes. A. Immunofluorescent staining for CCR2 (a marker of monocyte-derived macrophages) and CX3CR1 (a microglia marker) in glioma and normal brain tissues. Sections obtained from glioma surgical specimens and normal brains were immunostained using antibodies against CCR2 (green) and CX3CR1 (red) and then counterstained using DAPI (blue). **B.** Graphical analysis of (A) showing that the expression of CCR2+ cells in glioma tissues was much stronger than its expression in normal brain tissues. The macrophages in normal human brain tissues were CX3CR1+ and therefore microglial. The relative density of CCR2+ and CX3CR1+ cells were obtained from positive staining specimens.

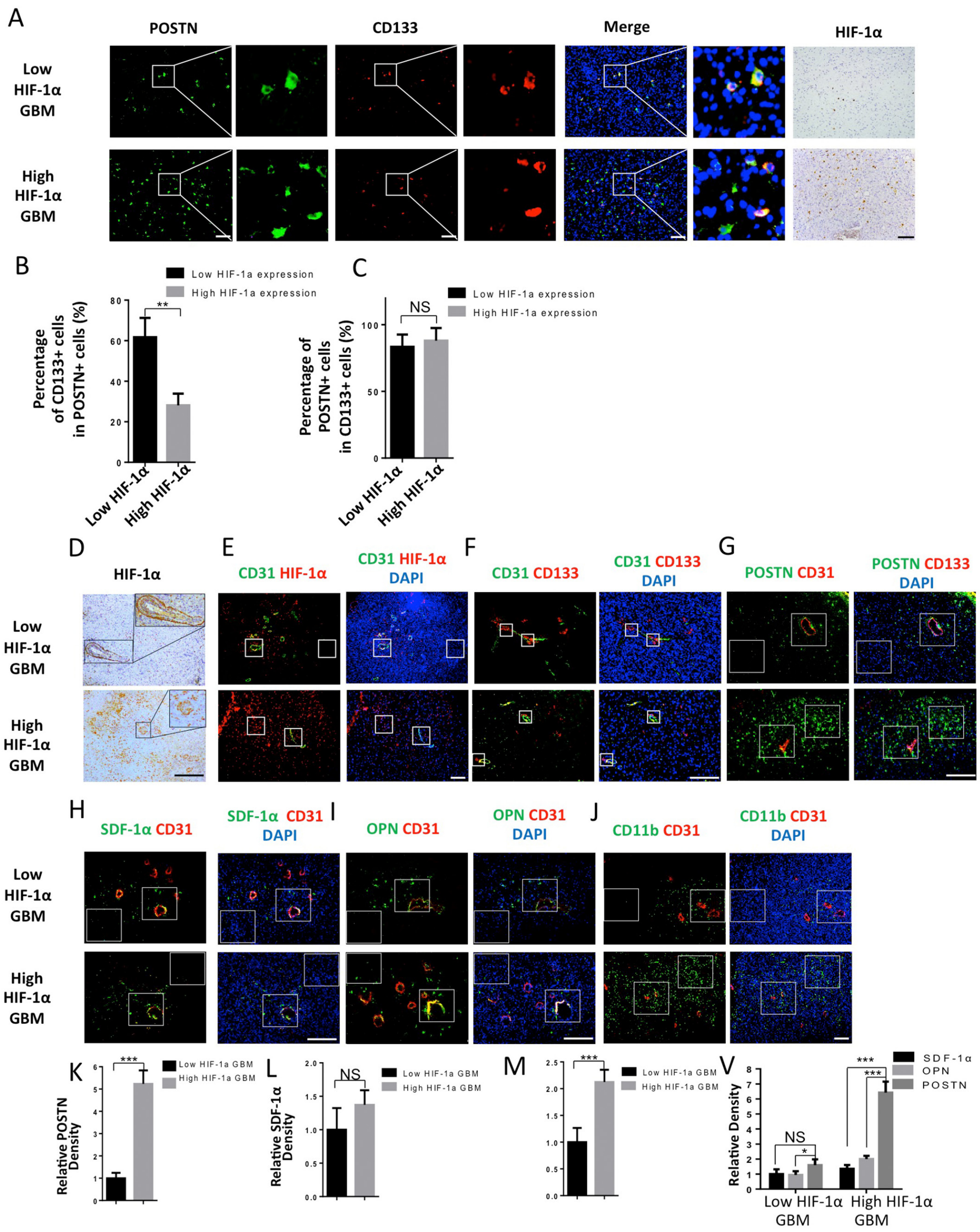

Supplementary Figure S3: The expression levels of POSTN, HIF-1α, CD133, SDF-1α, CD11b and OPN were associated with perivascular niches in both low and high HIF-1α expressing glioma tissues. A-C. Immunofluorescence staining for POSTN (green) and the GSLC marker CD133 (red) in human primary GBMs with different level of HIF-1α expression. Graphical analysis of (A) showing that POSTN was mainly expressed in GSLCs in low HIF-1α glioma tissue. **D** and **E.** High HIF-1α expression was observed in and around some CD31+ capillary walls in both the low and high hypoxic GBM tissues. **F.** Co-immunofluorescence analysis of CD133 and CD31 expression in sections obtained from low and high HIF-1α GBMs. **G.** POSTN and CD31 co-immunofluorescence in low and high HIF-1α GBM sections. **H.** SDF-1α and CD31 co-immunofluorescence in low and high HIF-1α GBM sections. SDF-1α was enriched in perivascular regions and increased in high HIF-1α glioma tissues. **I.** OPN and CD31 co-immunofluorescence in low and high HIF-1α GBM sections. OPN was enriched in perivascular regions and increased in high HIF-1α glioma tissues. **J.** The distribution of TAMs (CD11b+) was associated with capillaries. **K-M.** A graph describing the relative densities of POSTN, SDF-1α and OPN in low and high HIF-1α GBM sections. The positively stained and unstained cells (DAPI+) were then counted. Three specimens were selected from each group (low and high hypoxic glioma tissue) for the statistical analysis. **V.** The POSTN expression in high HIF-1α expressing glioma specimens was much higher than the expression of SDF-1α and OPN. The differences between the expression of POSTN and the other two chemotactic factors in low HIF-1α glioma specimens were much smaller than them in high HIF-1α glioma tissues. Scale bars, 100 μm. Protein and surface marker expressions were analyzed using ImageJ. *, P <0.05; **, P <0.01; ***, P <0.001; NS, P>0.05 (n =5 tumors; mean ± s.e.m.; two-tailed unpaired t-test).

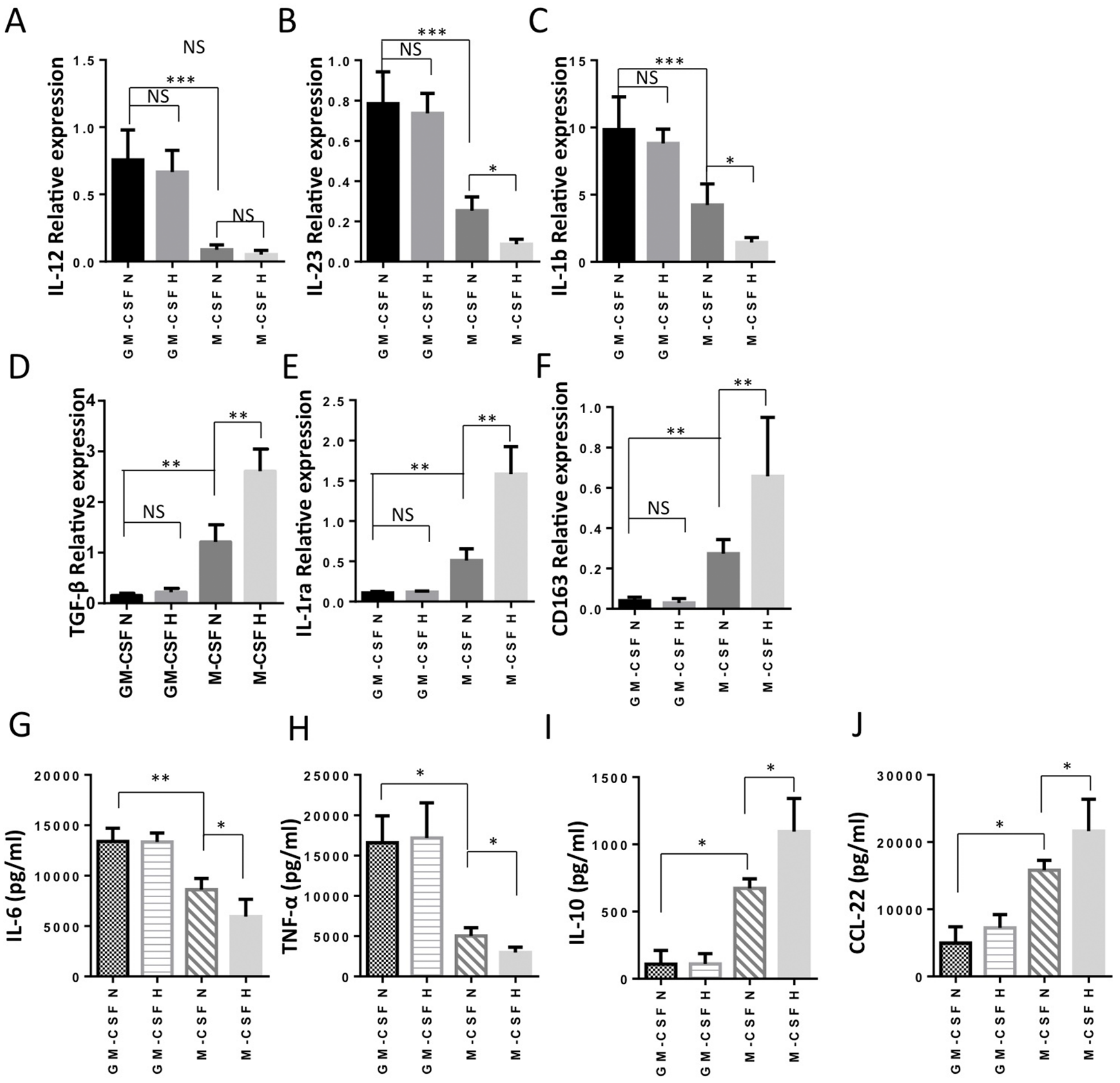

Supplementary Figure S4: Gene expression was analyzed in GM-CSF-induced macrophages, which were found to exhibit an M1 phenotype, while M-CSF-induced macrophages exhibited an M2 phenotype. A-F. The mRNA expression levels of IL-12, IL-23, IL-1b, TGF-β, IL-1ra and CD163 mRNA were differentially altered after monocytes were incubated with GM-CSF or M-CSF under normoxia (GM-CSF/M-CSF-N) or hypoxia (GM-CSF/M-CSF-H). **G-J.** The secretion of the cytokines IL-6, TNF-α, IL-10 and CCL-22 from monocytes was differentially altered after the cells were incubated with GM-CSF or M-CSF under normoxia (GM-CSF/M-CSF-N) or hypoxia (GM-CSF/M-CSF-H).

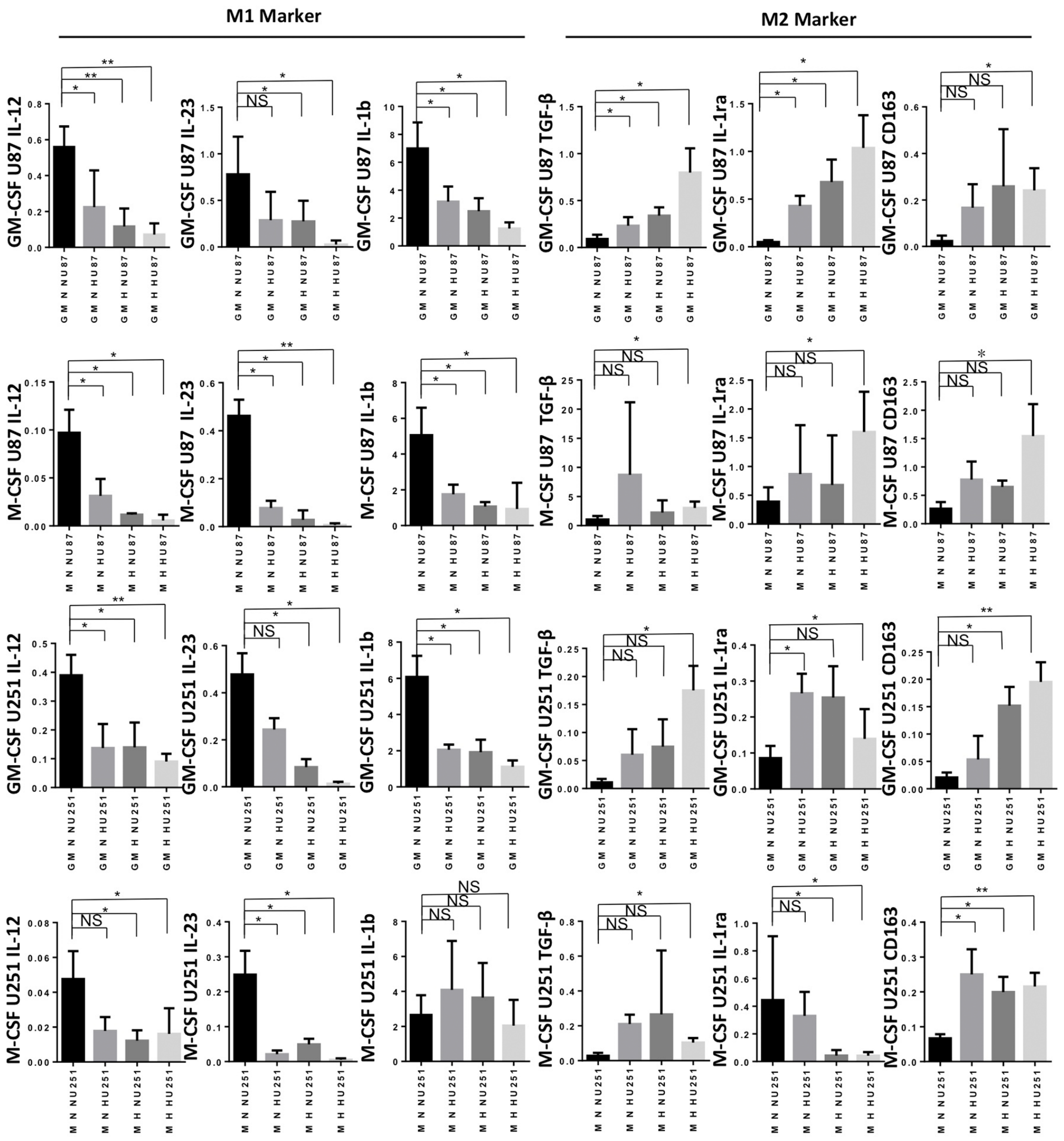

Supplementary Figure S5: Specific mRNA expression data for the heatmap shown in Figure 6.

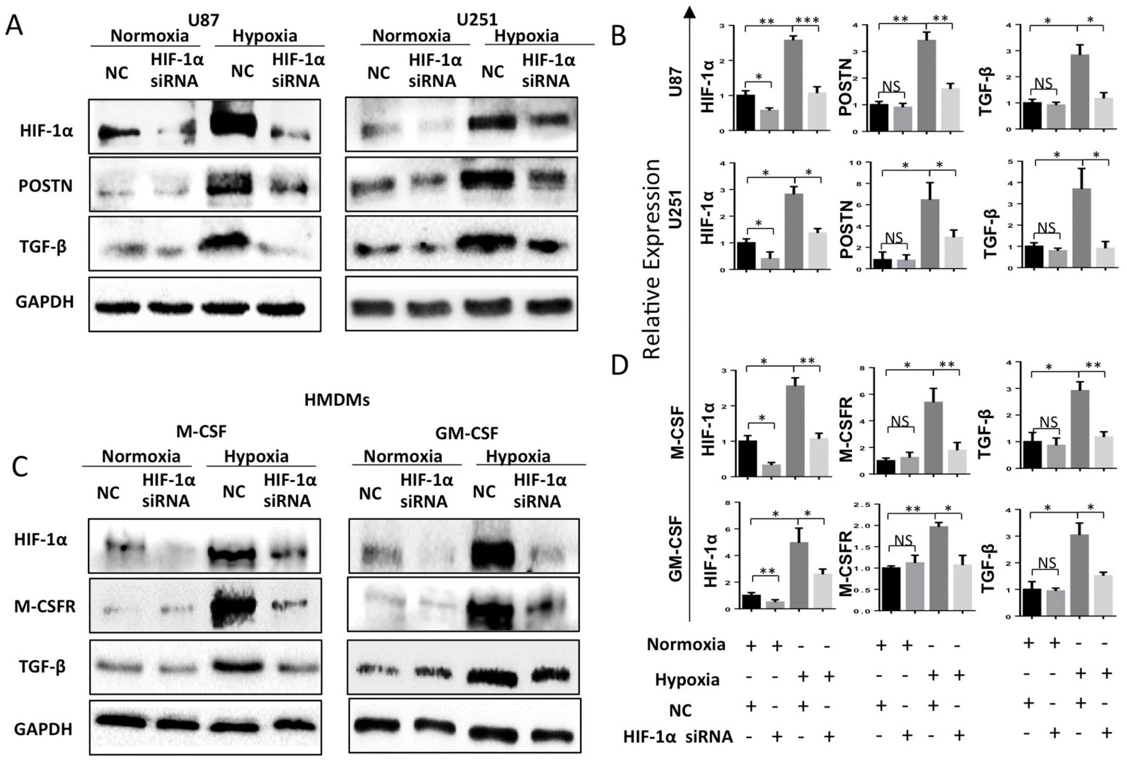

Supplementary Figure S6: Knockdown of Hif-1α inhibited the hypoxia-induced upregulation of POSTN, TGF-β and M-CSFR in U87/U251 cells and HMDMs. **A.** U87 and U251 cells were transfected with either 100 nM Hif-1α siRNA or nonspecific (NC) siRNA for 24 h, and the cells were then cultured while exposed to room air or under hypoxic conditions for 48 h. The cells were collected, and the cell lysates were obtained to detect Hif-1α, POSTN, TGF-β and GAPDH levels using western blot analysis. **B.** The densitometric analysis was performed using pooled data from three experiments. **C.** PBMCs were collected and stimulated with either GM-CSF or M-CSF for 48 h. The transformed macrophages were then transfected with either 100 nM Hif-1α siRNA or nonspecific (NS) siRNA for 8 h and then cultured while exposed to room air or under hypoxic conditions for 48 h. The cells were then collected, and cell lysates were obtained to detect the levels of Hif-1α, TGF-β, M-CSFR and GAPDH using western blot analysis. **D.** The densitometric analysis was performed using pooled data from three experiments. The protein expression was analyzed using ImageJ. *, $P < 0.05$; **, $P < 0.01$; ***, $P < 0.001$; NS, $P > 0.05$ ($n = 5$ tumors; mean ± s.e.m.; two-tailed unpaired t-test).

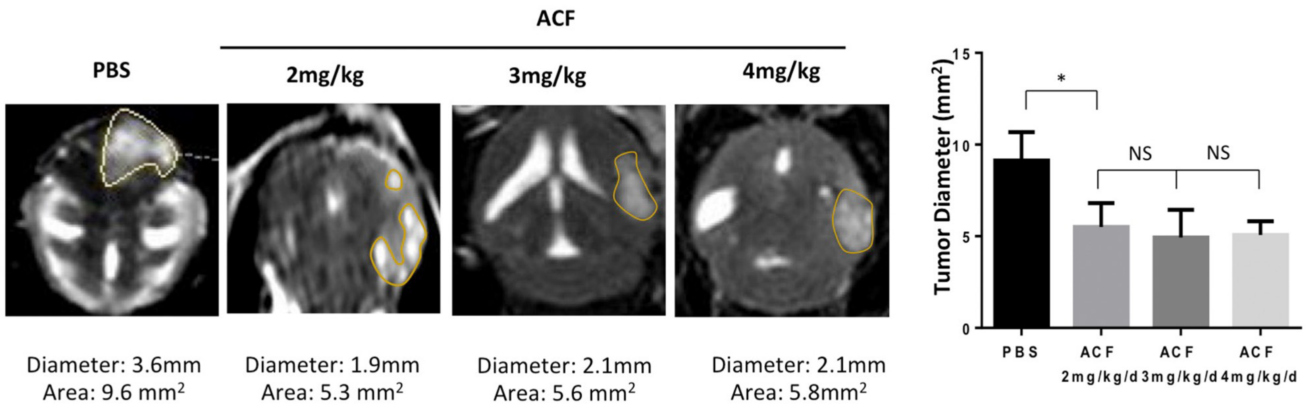

Supplementary Figure S7: MRI scan images obtained on day 20. The results demonstrated that the glioma size was smaller in the ACF treatment group than in the PBS treatment group. However, the difference in tumor size between the 3 groups that were administered different doses of ACF were not prominent at 20 days after implantation.

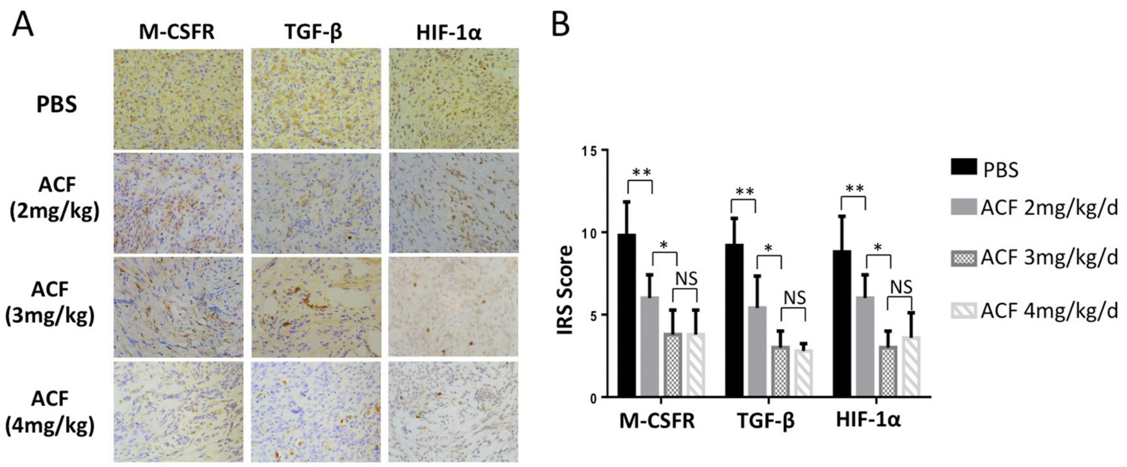

Supplementary Figure S8: M-CSFR, TGF-β and HIF-1α staining in consecutive tissue slides that were obtained from PBS- and ACF-treated tumors. The expression levels of these receptors and proteins were lower after treatment.

Supplementary Table S1: The demographic parameters of the patients who participated in the study

Enter		42	
Gender			
	Male	12	28.57%
	Female	30	71.43%
Age(years)			
	Average(range)	51.17(4-75)	
Pathological type			
	Astrocytoma	8	19.05%
	Anaplastic astrocytoma	9	21.43%
	Pilocytic astrocytoma	3	7.14%
	Oligodendroglioma	1	2.38%
	Anaplastic oligodendroglioma	2	4.76%
	Glioblastoma	20	47.62%
	Dysembryoplastic neuroepithelial tumor	1	2.38%
WHO tumor grade at diagnosis			
	I	2	4.76%
	II	9	21.43%
	III	11	26.19%
	IV	20	69.05%

Supplementary Table S2: The sequences of the primers used for qRT-PCR

Protein	5'primer	3'primer
**IL-12 40p**	CCA GAG CAG TGA GGT CTT AGG C	TGT GAA GCA GCA GGA GCG
**IL-23p19**	TCC CTA CTA GGA CTC AGCC AAC	GCT GCC ACT GCT GAC TAG AA
**IL-1b**	AGC TAC GAA TCT CCG ACC AC	CGT TAT CCC ATG TGT CGA AGA A
**TGF-b**	CAA TTC CTG GCG ATA CCT CAG	GCA CAA CTC CGG TGA CAT CAA
**IL-1ra**	GAG GGA AGA TGT GCC TGT CC	CCA CTG TCT GAG CGG ATG AA
**CD163**	GAC GCA TTT GGA TCA TGT	CCC ACC GTC CTT GGA ATT TGA
**PGK-1**	TGG ACG TTA AAG GGA AGC GG	GCT CAT AAG GAC TAC CGA CTT GG
**GAPDH**	GCA CCG TCA AGG CTG AGA AC	TGG TGA AGA CGC CAG TGG A
